# Dichloroacetate and Pyruvate Metabolism: Pyruvate Dehydrogenase Kinases as Targets Worth Investigating for Effective Therapy of Toxoplasmosis

**DOI:** 10.1128/mSphere.01002-20

**Published:** 2021-01-06

**Authors:** Mariana Galvão Ferrarini, Lindice Mitie Nisimura, Richard Marcel Bruno Moreira Girard, Mayke Bezerra Alencar, Mariana Sayuri Ishikawa Fragoso, Carlla Assis Araújo-Silva, Alan de Almeida Veiga, Ana Paula Ressetti Abud, Sheila Cristina Nardelli, Rossiane C. Vommaro, Ariel Mariano Silber, Marie France-Sagot, Andréa Rodrigues Ávila

**Affiliations:** a Laboratoire de Biométrie et Biologie Évolutive, UMR 5558, CNRS, Université de Lyon 1, Villeurbanne, France; b Laboratório de Pesquisa em Apicomplexa, Instituto Carlos Chagas, Fiocruz, Paraná, Brazil; c Laboratory of Biochemistry of Tryps, Department of Parasitology, Institute of Biomedical Sciences, University of São Paulo, São Paulo, SP, Brazil; d Laboratório de Ultraestrutura Celular Hertha Meyer, Instituto de Biofísica Carlos Chagas Filho, Universidade Federal do Rio de Janeiro, Rio de Janeiro, Brazil; e Instituto Nacional de Ciência e Tecnologia em Biologia Estrutural e Bioimagens, Universidade Federal do Rio de Janeiro, Rio de Janeiro, Brazil; f INRIA Grenoble Rhône-Alpes, Montbonnot-Saint-Martin, France; University at Buffalo

**Keywords:** toxoplasmosis, DCA, metabolism

## Abstract

Toxoplasmosis, a protozoan infection caused by Toxoplasma gondii, is estimated to affect around 2.5 billion people worldwide. Nevertheless, the side effects of drugs combined with the long period of therapy usually result in discontinuation of the treatment. New therapies should be developed by exploring peculiarities of the parasite’s metabolic pathways, similarly to what has been well described in cancer cell metabolism. An example is the switch in the metabolism of cancer that blocks the conversion of pyruvate into acetyl coenzyme A in mitochondria. In this context, dichloroacetate (DCA) is an anticancer drug that reverts the tumor proliferation by inhibiting the enzymes responsible for this switch: the pyruvate dehydrogenase kinases (PDKs). DCA has also been used in the treatment of certain symptoms of malaria; however, there is no evidence of how this drug affects apicomplexan species. In this paper, we studied the metabolism of T. gondii and demonstrate that DCA also inhibits T. gondii’s *in vitro* infection with no toxic effects on host cells. DCA caused an increase in the activity of pyruvate dehydrogenase followed by an unbalanced mitochondrial activity. We also observed morphological alterations frequently in mitochondria and in a few apicoplasts, essential organelles for parasite survival. To date, the kinases that potentially regulate the activity of pyruvate metabolism in both organelles have never been described. Here, we confirmed the presence in the genome of two putative kinases (T. gondii PDK [TgPDK] and T. gondii branched-chain α-keto acid dehydrogenase kinase [TgBCKDK]), verified their cellular localization in the mitochondrion, and provided *in silico* data suggesting that they are potential targets of DCA.

**IMPORTANCE** Currently, the drugs used for toxoplasmosis have severe toxicity to human cells, and the treatment still lacks effective and safer alternatives. The search for novel drug targets is timely. We report here that the treatment of T. gondii with an anticancer drug, dichloroacetate (DCA), was effective in decreasing *in vitro* infection without toxicity to human cells. It is known that PDK is the main target of DCA in mammals, and this inactivation increases the conversion of pyruvate into acetyl coenzyme A and reverts the proliferation of tumor cells. Moreover, we verified the mitochondrial localization of two kinases that possibly regulate the activity of pyruvate metabolism in T. gondii, which has never been studied. DCA increased pyruvate dehydrogenase (PDH) activity in T. gondii, followed by an unbalanced mitochondrial activity, in a manner similar to what was previously observed in cancer cells. Thus, we propose the conserved kinases as potential regulators of pyruvate metabolism and interesting targets for new therapies.

## Abstract (precis)

Currently, the drugs used for toxoplasmosis have severe toxicity to human cells, and the treatment still lacks effective and safer alternatives. The search for novel drug targets is timely.

## INTRODUCTION

Apicomplexa comprise a vast group of unicellular parasites that are causative agents of human and animal diseases with a huge impact on world populations and economy. Distinctive features of Apicomplexa include a specialized apical invasion machinery, termed the apical complex, and most species bear a relict plastid derived from a red algal secondary endosymbiont known as the apicoplast (1, 2). Apicomplexans, such as *Plasmodium* spp., account for up to 500 million clinical cases of malaria each year, with up to 2 million deaths, while Toxoplasma gondii infects approximately one-third of the human population (3, 4). Moreover, the burden of clinical toxoplasmosis in humans is considered to be higher in Latin America than in Europe and North America (5, 6). Active infection during the acute phase is characterized by the presence of tachyzoites, whereas the presence of tissue cysts characterizes the latent disease. The acute phase of toxoplasmosis is usually asymptomatic, and treatment is not necessary. However, severe clinical symptoms occur in immunosuppressed patients and may be associated with a clinical manifestation of congenital and ocular toxoplasmosis (7–10).

The recommended first-line treatment of the infection is a combination of pyrimethamine and sulfadiazine, which inhibits the folate pathway and consequently the proliferation and survival of the parasites (11). However, those drugs are also toxic for human cells, and significant adverse effects of pyrimethamine have been reported for different clinical manifestations of the disease (12); in rare cases, the treatment may even be fatal (13). Alternative drugs can replace the first-line ones, but the treatment is less effective (14, 15). Moreover, prolonged courses of therapy are required for the treatment and suppression of the infection, and current medicines are unable to treat the tissue cyst stage of T. gondii. All together, the adverse effects and the ineffectiveness of the prolonged therapy at the chronic phase usually result in discontinuation of the therapy and consequently impact the treatment outcome.

The challenge toward new drugs includes finding chemical entities with diminished toxicity, usable in short-term treatment schemes and able to eliminate tissue cysts. The improvement of the existing therapies for toxoplasmosis is essential and urgent. Improved therapies can emerge from different strategies and can be inspired by the solutions found against other diseases. For example, cancer treatment faces the lack of specific proteins of cancer cells since they have the same genetic background as the healthy cells; similarly, the treatment of eukaryotic pathogens may face the difficulty of finding specific proteins which do not bear similarity to human proteins (16). In this sense, the search for antitumor drugs has also focused on understanding the differential metabolism of tumors. The main strategy is to look for targets that are part of metabolic pathways that can be affected without causing collateral damage to the healthy cells, which also potentially have the same target proteins (17). An example of this is the Warburg effect, a milestone of most cancer cells (and proliferative tissues as well) which can be described as a metabolic switch in the production of ATP by increasing the rate of glycolysis, followed by lactic acid fermentation in the cytosol (even in the presence of oxygen), rather than maintenance of a low rate of glycolysis, followed by oxidation of pyruvate in mitochondria, as is observed in most noncancer cells (18, 19). This effect is usually caused by the overexpression of an enzyme called pyruvate dehydrogenase kinase (PDK), which blocks the action of the pyruvate dehydrogenase (PDH) complex, thus making these cells incapable of converting pyruvate into acetyl coenzyme A (AcCoA) inside the mitochondrion (20). All together, these changes create an antiapoptotic and proliferative environment essential for tumors to thrive (21).

Dichloroacetic acid, or dichloroacetate (DCA), is a drug capable of inactivating PDK, restoring the normal oxidation of pyruvate into AcCoA in mitochondria and inducing both apoptosis and the inhibition of tumor growth (22). The PDK/PDH axis became a therapeutic target in cancer, and DCA has been implicated in the successful treatment of cancer cells while allowing normal cells to survive (23, 24). Following a similar rationale, DCA has also been used in the treatment of lactic acidosis in malaria, a disease caused by *Toxoplasma*’s closely related pathogens from the genus *Plasmodium* (25–27). A recent study has also verified a parasite-killing activity by DCA on T. gondii, Plasmodium falciparum, and other protozoan parasites (28). However, it is not clear how the treatment directly affects the metabolism of such parasites. This is a good example of how understanding the metabolism of such parasites as well as exploiting their vulnerabilities can reveal novel candidate drug targets (29).

In this work, through biochemistry and cellular biology approaches, we confirmed that DCA indeed affects the metabolism and morphology of T. gondii, impairing the *in vitro* infection without toxic effects to mammalian cells. We coupled these results with computational biology analyses along with subcellular localization experiments to propose two mitochondrial kinase enzymes (T. gondii PDK [TgPDK] and T. gondii branched-chain α-keto acid dehydrogenase kinase [TgBCKDK]) involved in the regulation of pyruvate metabolism as possible targets for the action of this drug in this parasite. Based on our results, we discuss the use of DCA to investigate the regulation of PDH activity in order to propose new perspectives in the treatment of human toxoplasmosis.

## RESULTS

### DCA treatment does not cause cytotoxic effects in human cells but impairs the parasite infection.

**FIG 1:**
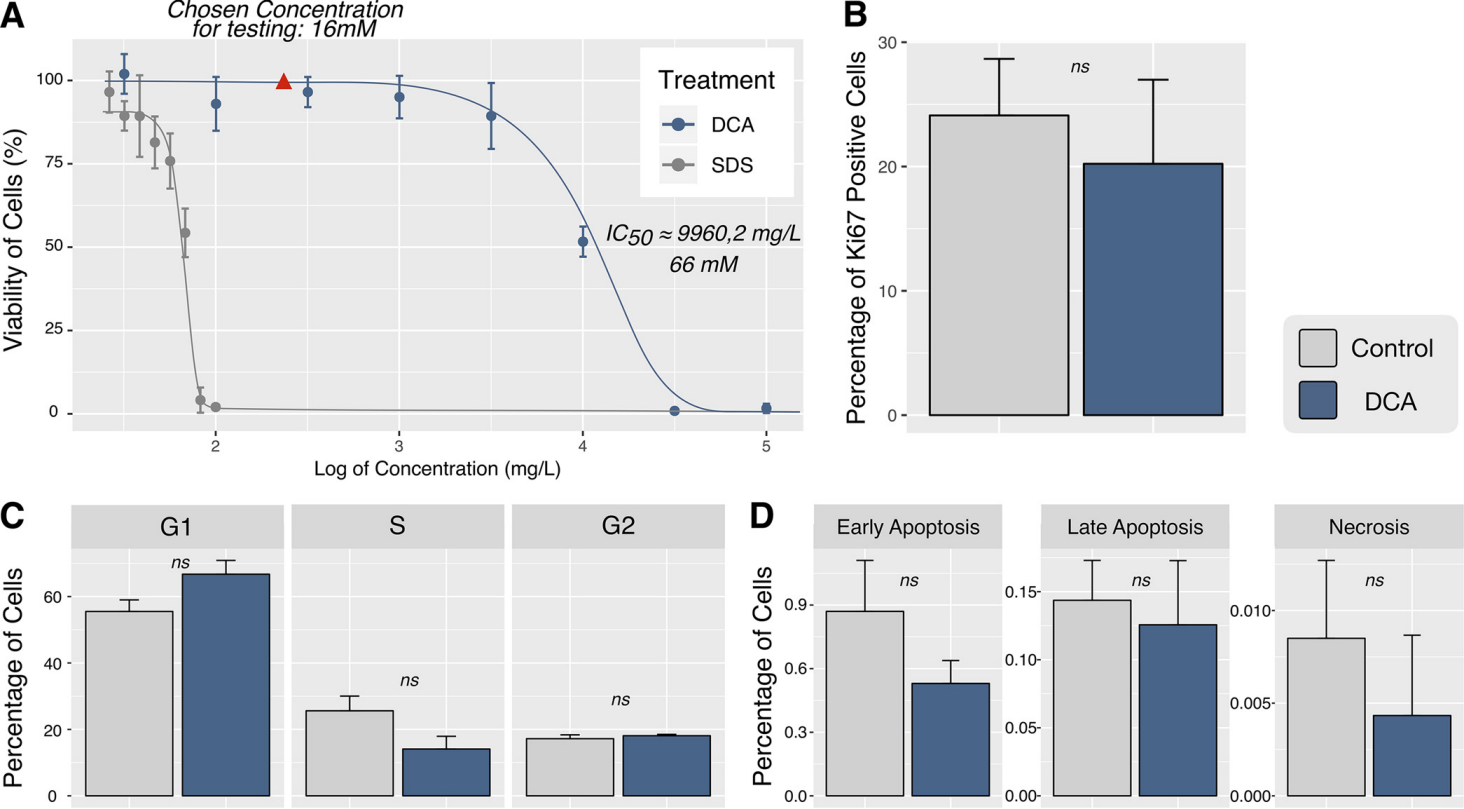
DCA does not cause toxicity effects in NHDF. (A) Dose-response graph for NHDF treated with SDS (gray) or DCA (blue). The IC_50_ of DCA was calculated as 2 orders of magnitude lower than that of SDS (which was used as a positive control for toxicity [Table S3 in the supplemental material]). Based on the curve, the optimal concentration of 16 mM was chosen to guarantee that 100% of cells remained viable. (B) The proliferation assay was performed by quantification of NHDF positive for staining of the Ki67 protein using 16 mM DCA. (C) The percentages of NHDF at different phases of the cell cycle were evaluated based on propidium iodide staining, with NHDF treated (blue) or not treated with DCA (gray) at 16 mM. (D) Cells in early apoptosis, late apoptosis, and necrosis were observed by the apoptosis detection probe annexin V and the necrosis marker 7-aminoactinomycin D (7-AAD). The lack of statistical significance between the negative control and the DCA treatments (ns) was revealed by Student’s *t* tests between conditions. Data are presented as bar plots of results from three independent experiments.

We used primary cell cultures of normal human dermal fibroblasts (NHDF) to grow T. gondii tachyzoites. For this reason, we initially evaluated DCA cytotoxicity in these cells using the neutral red uptake assay based on OECD 129 guidelines (30). We defined optimal DCA concentrations based on a dose-response graph (Fig. 1A). The half-maximal inhibitory concentration (IC_50_) of DCA was calculated from Hill’s function using its concentration values, while the median lethal dose (LD_50_) was calculated using the formula log LD_50_ (millimoles per kilogram of body weight) = 0.439 log IC_50_ (millimolar) + 0.621 (*R*^2^ = 0.452), according to the Globally Harmonized System (GHS) for the classification of chemicals that cause acute toxicity (30). The GHS is a chemical classification and labeling standardization system and has five categories for acute toxicity, according to LD_50_ chemical values (31). With an IC_50_ of 9,920.7 μg/ml and an LD_50_ of 3,620.6 (mg/kg), DCA is classified in category 5, with a low acute toxicity hazard (32, 33) (see Table S1 in the supplemental material).

10.1128/mSphere.01002-20.7 TABLE S1 IC_50_ calculations of DCA versus SDS, used as a positive toxicity control. Download Table S1, XLSX file, 0.04 MB. Copyright © 2021 Ferrarini et al. 2021 Ferrarini et al. This content is distributed under the terms of the Creative Commons Attribution 4.0 International license.

In order to evaluate other parameters of DCA cytotoxicity, we chose the DCA concentration of 16 mM, which corresponds to a point in the curve for which we can guarantee that cell viability is 100% (Fig. 1A, red arrowhead). Furthermore, we show that the chosen concentration did not affect NHDF proliferation (Fig. 1B) or the cell cycle (Fig. 1C) and did not induce apoptosis or necrosis (Fig. 1D). Based on the parameters measured here, DCA at a concentration of 16 mM did not cause any cytotoxic effects to NHDF.

**FIG 2:**
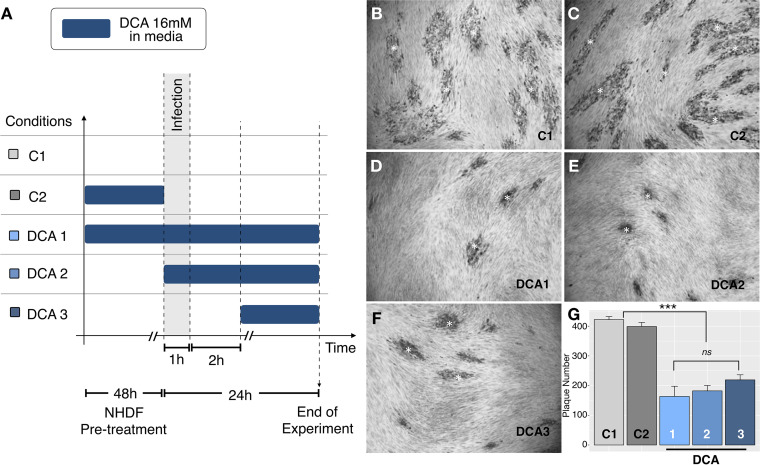
DCA impairs the growth of T. gondii RH strains in a plaque assay. (A) All treatments tested; (B) no DCA pretreatment of NHDF plus RH tachyzoites (C1); (C) NHDF pretreated with DCA for 48 h with RH tachyzoites (C2); (D) NHDF pretreated with DCA for 48 h with RH tachyzoites and 16 mM DCA (DCA 1); (E) no DCA pretreatment plus RH tachyzoites plus 16 mM DCA (DCA 2); (F) no DCA pretreatment plus RH tachyzoites plus 16 mM DCA 2 h postinvasion (DCA 3); (G) quantification of the number of plaques (* in panels B to F). The data are means ± standard errors of the means (SEM) of results from three independent experiments. Significance was tested with a one-way ANOVA and a Tukey posttest (***, *P* < 0.001).

After establishing this optimal concentration for the cells, we then evaluated whether treatment with DCA would affect the multiplication of T. gondii using an *in vitro* assay (Fig. 2). In this assay, parasite multiplication causes cytolytic damage, creating visible holes (plaques) in the NHDF monolayer. We tested 5 different conditions to quantify the number of plaques after 7 days of parasite infection in both the presence and absence of DCA. These conditions are summarized in Fig. 2A and included 2 negative controls along with 3 different DCA treatments. For control 1 (C1), untreated cells were infected with tachyzoites without any addition of DCA in the medium; for control 2 (C2), cells were pretreated with DCA for 48 h, washed (no DCA was further added to the medium), and infected with tachyzoites. These different controls were necessary to show that the DCA effect was not the result of a change in the host cell (indirect effect), as opposed to DCA having a direct effect on the parasites.

As shown in Fig. 2G, the number of plaques decreased similarly and significantly (*P *< 0.001) under all conditions tested for DCA treatment. The different DCA treatments were as follows: cells were pretreated for 48 h with DCA, infected with tachyzoites, and maintained with DCA in the medium until the end of the analysis (DCA 1); untreated cells were infected with tachyzoites and treated with DCA until the end of the analysis (DCA 2); and cells were infected with tachyzoites, and DCA was added only after 2 h of established infection (DCA 3). Note that even when DCA was added after the parasites invaded the cells (DCA 3), we observed similar effects (Fig. 2G).

**FIG 3:**
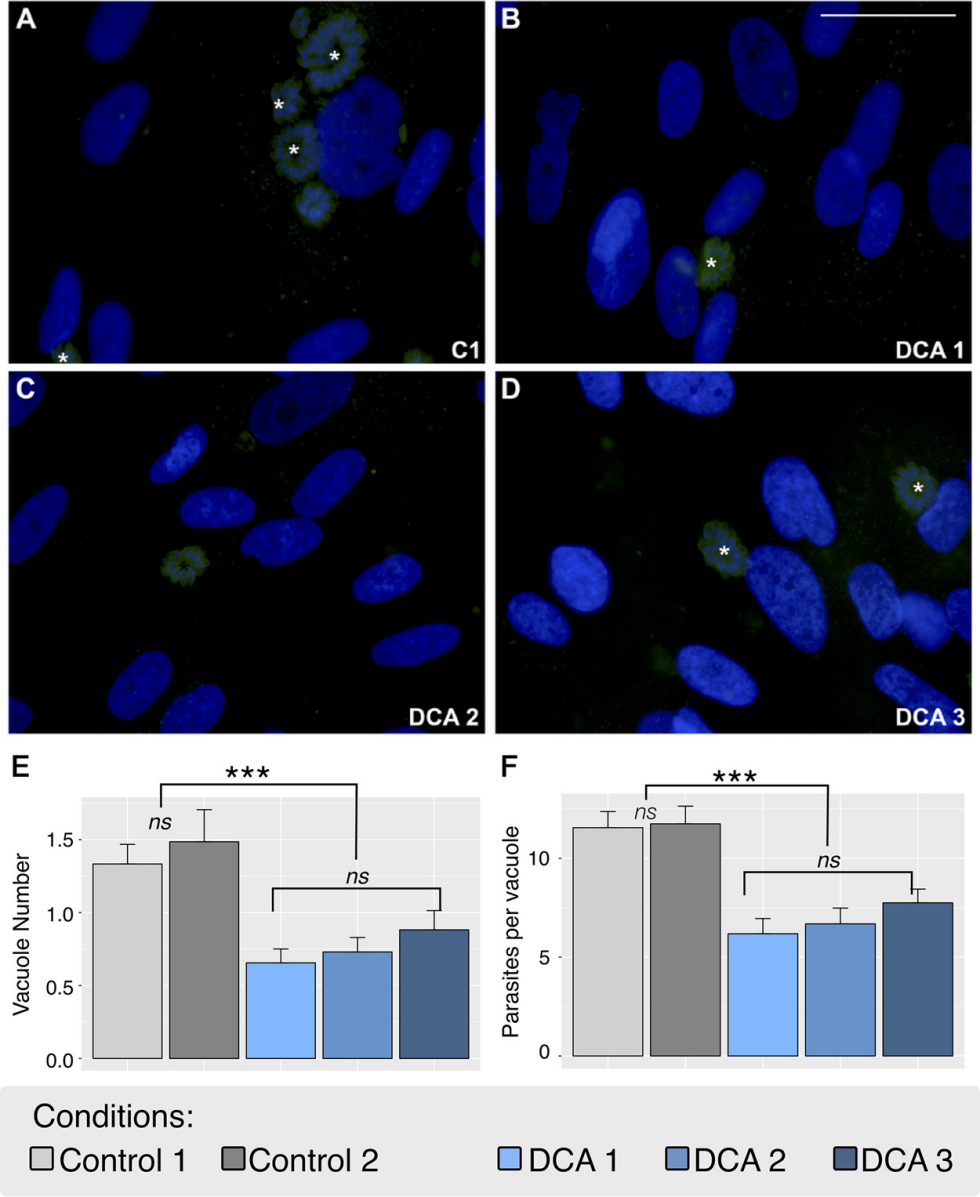
DCA treatment influence on T. gondii invasion and replication. *In vitro* immunofluorescence assays show parasites in green and the nuclei of cells in blue. (A) Control 1 (C1); (B) DCA 1; (C) DCA 2; (D) DCA 3. Control 2 is not shown in the image, but the result was included in the quantification graph. (E) Vacuole number (* in panels A to D) per quantification field; (F) parasite number per vacuole quantification. The data are means ± SEM from three independent experiments. Scale bar = 50 μM. Significance was tested with a one-way ANOVA and a Tukey posttest (***, *P* < 0.001).

However, the plaque assay could not distinguish if DCA affected the replication of the parasites or their ability to invade the cells (or both). Thus, we further performed an immunofluorescence assay to quantify distinctively the number of parasite vacuoles as well as the number of parasites per vacuole to confirm an effect on the proliferation of the parasites (Fig. 3). Interestingly, the DCA treatment significantly reduced (*P* < 0.001) both the number of vacuoles (Fig. 3E) and the number of parasites inside the vacuoles (Fig. 3F) in all DCA treatments. As a control for all the assays, we treated the cells with DCA prior to infection (control 1). As explained above, this was performed to verify that the efficacy of the treatment was not confounded by a shift in NHDF metabolism. Nevertheless, the pretreatment of NHDF with DCA did not alter the infection, invasion, or replication of T. gondii (Fig. 3A). This confirmed our initial assumption that DCA had a direct action on the parasites while keeping the host cells functional and unharmed.

### DCA treatment triggered an increase of PDH activity associated with an imbalance of mitochondrial activities.

**FIG 4:**
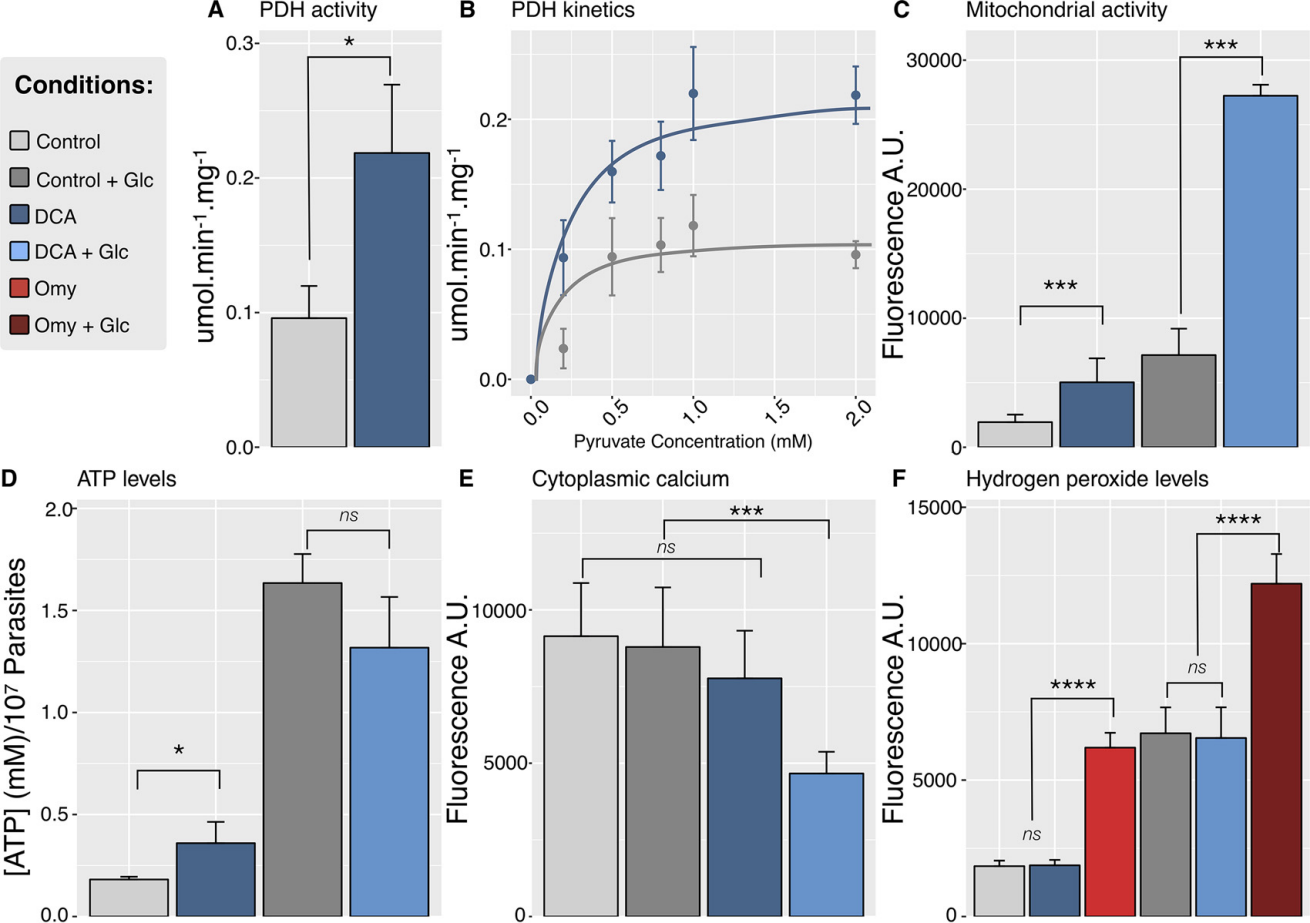
Evaluation of mitochondrial redox capacity. (A) The PDH activity was measured in cell extract previously treated or not treated with 16 mM DCA in the presence of 2 mM pyruvate. (B) PDH activity was measured in T. gondii cell extract previously treated or not treated with 16 mM DCA in the presence of various concentrations of pyruvate. (C) Mitochondrial activity using the resazurin assay. (D) Intracellular ATP levels. (E) Cytoplasmic Ca^2+^ level. (F) H_2_O_2_ levels. The cells were treated or not treated with glucose and/or DCA and are named, respectively, DCA Glc, Glc, DCA, and Control. As a positive control of H_2_O_2_ production, 5 μg/ml oligomycin (Omy) was used. The data correspond to three independent biological experiments. One-way ANOVA followed by a Tukey posttest was used for statistical analysis to compare the values to the respective control in panels E and F, and a *t* test was used on the data shown in panels C and D (****, *P* < 0.0001; ***, *P* < 0.01; *, *P* < 0.05). A.U., arbitrary units.

Unlike other eukaryotes, T. gondii possesses a PDH located in the apicoplast and not in the mitochondrion (34). Indeed, a structurally related mitochondrial enzyme complex called branched-chain α-keto acid dehydrogenase (BCKDH), usually involved in the degradation of branched-chain amino acids (BCAA), was detected in the mitochondrion and was described to have a major PDH activity in T. gondii (35). Since DCA treatment has been shown to increase PDH activity in cancer cells, we used protein extracts of T. gondii previously treated or not with DCA to measure the global PDH activity (Fig. 4A). Our data show that T. gondii treated with DCA exhibits a significant increase (*P *< 0.05) in PDH activity. We then proceeded to evaluate whether the PDH activity was dependent on the pyruvate concentration, which is the substrate of PDH (Fig. 4B). Under both conditions, the kinetics was compatible with a Michaelis-Menten hyperbolic function (Fig. 4B), which allowed us to compute a *K_m_* of 0.1374 ± 0.1308 mM and a maximum rate of metabolism (*V*_max_) of 0.1188 ± 0.02008 μmol · min^−1^ · mg^−1^ for untreated parasites and a *K_m_* of 0.1439 ± 0.06756 mM and a *V*_max_ of 0.2293 ± 0.01963 μmol · min^−1^ · mg^−1^ for DCA-treated parasites. These data indicate that the *V*_max_ in DCA-treated T. gondii significantly increased, while the *K_m_* value remained constant. Remarkably, to our knowledge, this is the first time that the PDH kinetic parameters are reported for T. gondii. Interestingly, in humans, two isoforms of the PDH complex are present in different tissues (PDH1 and PDH2). PDH2 has a higher affinity for pyruvate (*K_m_* = 0.0359 mM), while PDH1 has a lower affinity (*K_m_* = 0.0648 mM), and according to Korotchkina et al. (36), the *V*_max_ depends on the expression levels in each tissue. Since we showed that PDH activity not only was present but also increased in DCA-treated parasites, we were interested in evaluating the possible interference of DCA in mitochondrial metabolism. DCA-treated T. gondii organisms were submitted to a resazurin-based assay to determine its mitochondrial oxido-reductive capacity (Fig. 4C). The parasites were also incubated with 5 mM glucose to induce glycolysis. DCA-treated parasites exhibited a significant increase of their mitochondrial activity compared to that of the negative controls. The treatment with DCA in the presence of glucose showed the highest mitochondrial activity, which is consistent with the previously observed increase of PDH activity in DCA-treated parasites (Fig. 4C).

As we evidenced a mitochondrial functionality alteration in DCA-treated parasites, we also investigated whether other associated mitochondrial activities, such as the production of ATP, the level of cytoplasmic Ca^2+^, and the level of reactive oxygen species (ROS), were altered. Parasites were incubated (or not) with DCA in the presence or absence of 5 mM glucose, and intracellular ATP was measured by a luciferase assay (Fig. 4D). In the presence of glucose, DCA-treated parasites showed no significant increase in their intracellular amounts of ATP; however, in the absence of glucose, we detected a significant difference (*P *< 0.05) in the intracellular levels of ATP. To determine if the DCA treatment could trigger changes in cytosolic Ca^2+^ levels, DCA-treated parasites (in the presence or absence of glucose) were incubated with Fluo-4 AM and analyzed by fluorometry (Fig. 4E). Treated parasites exhibited a decrease in cytosolic Ca^2+^ levels under both conditions. Interestingly, the increase of PDH activity triggered by DCA was not followed by an increase in ROS production (Fig. 4F). Furthermore, we were not able to detect any specific BCKDH activity (Fig. S1) from the BCAA degradation pathway. This is in accordance with previous results (35) showing that BCKDH possesses essentially PDH activity in this parasite. Taken together, these data point to the fact that DCA increases the global PDH activity, triggering deregulation of the mitochondrial function, which is evidenced by alterations in cytosolic Ca^2+^ homeostasis, mitochondrial redox activities, and ATP production.

10.1128/mSphere.01002-20.1 FIG S1 BCKDH activity in T. gondii and Trypanosoma cruzi. The activity was measured in cell-free extracts of T. gondii. As a positive control, we measured the BCKDH activity in extracts of T. cruzi epimastigotes. In both cases, reaction mixtures containing 15 mM HEPES, 0.1 mM CoA-SH, 0.2 mM TPP, 2.5 mM NAD^+^, 3 mM MgCl_2_ at pH 7.2, and 0.5 mM 4-methyl-2-oxovaleric acid were prepared. The measurements are expressed as relative activities (percentages). ND, not detected. Download FIG S1, PDF file, 0.1 MB. Copyright © 2021 Ferrarini et al. 2021 Ferrarini et al. This content is distributed under the terms of the Creative Commons Attribution 4.0 International license.

### DCA has a prominent effect on mitochondrion morphology.

**FIG 5:**
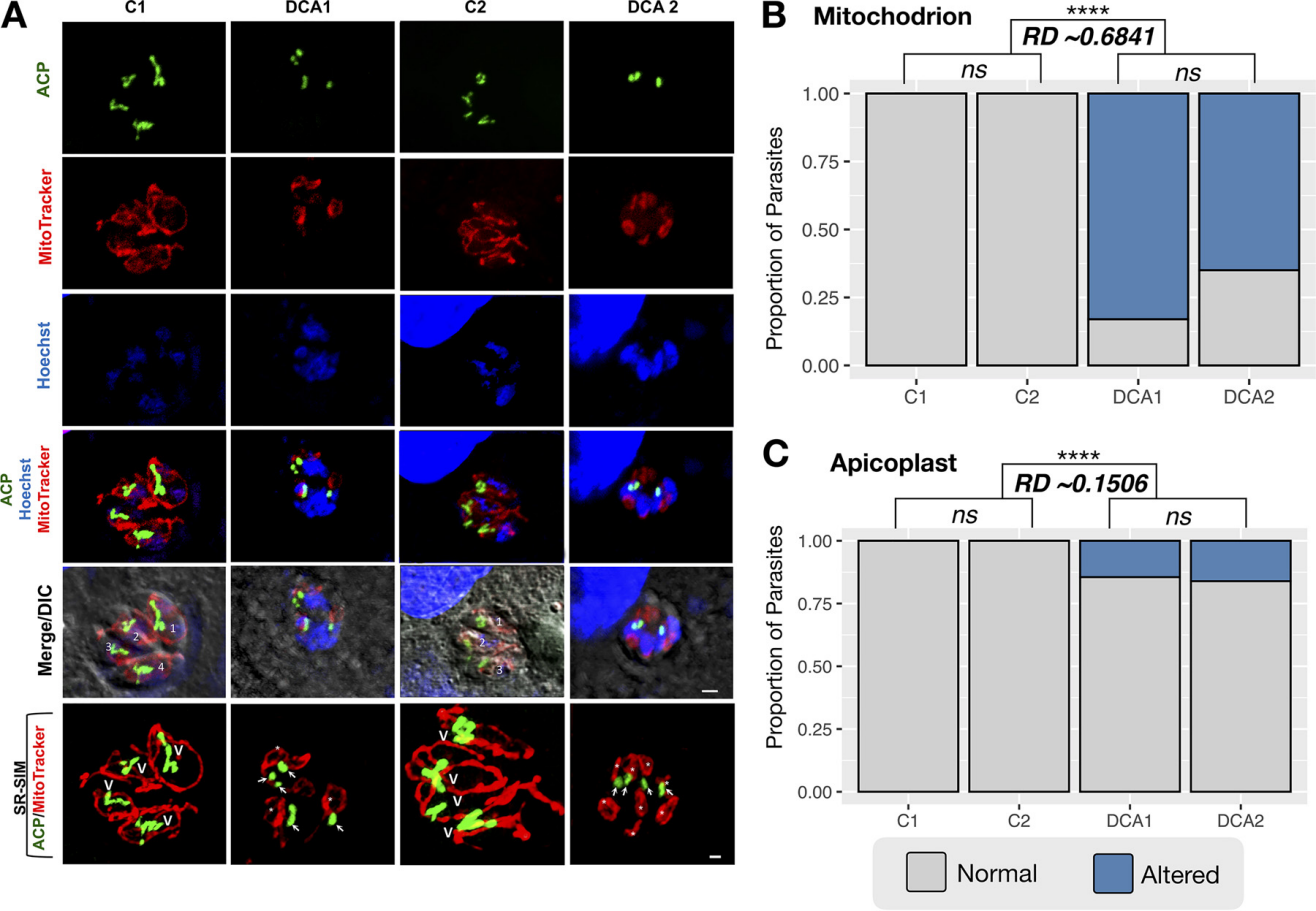
DCA effect on T. gondii endodyogeny. (A) High-resolution microscopy analysis of NHDF infected with RH-ACP-YFP tachyzoites. As explained for Fig. 2A, control 1 (C1) consisted of infected NHDF without any treatment; control 2 (C2) depicts NHDF pretreated with DCA for 48 h and then infected. DCA1, NHDF pretreated with DCA for 48 h, infected, and treated with DCA until the end of the experiment; DCA2, infected NHDF treated with DCA until the end of the experiment. Apicoplasts (ACP) are in green, mitochondria (MitoTracker) are in red, and nuclei (Hoechst dye) are in blue. LSM module images, scale bar = 2 μm; SR-SIM images, scale bar = 1 μm. White numbers, the formation of large atypical masses of damaged parasites seen as large nondividing nuclei; v, the typical V shape of apicoplasts; arrows, smaller mitochondria that may have originated from the fragmentation of the organelle; asterisks, fragments of apicoplasts. (B and C) The DCA effects were quantified in parasites per 100 cells. The results represent the average proportions between healthy and altered parasites from at least two independent experiments for each condition. Statistical analysis was performed with a Fisher test between DCA-treated and control conditions. ****, *P* < 0.0001. The effect size of the drug was measured as the risk difference (RD) (Table S3 in the supplemental material).

Since we were not able to distinguish biochemically if the increase of global PDH activity after DCA treatment was due to alterations of either BCKDH (present in mitochondria) or PDH (prevalent in apicoplasts) activity, we decided to perform high-resolution fluorescence microscopy in order to observe how DCA treatment affected either the mitochondrion or the apicoplast organelles. As shown in Fig. 5, we observed the presence of typical rosettes of parasites in the control samples (C1 and C2), each parasite had a single mitochondrion, one apicoplast and one nucleus, all correctly positioned. Tachyzoites appeared individualized in the parasitophorous vacuole; T. gondii mitochondria had the regular lasso shape, and apicoplasts presented the typical V shape (white “v” in Fig. 5A) in the course of the endodyogeny process. In accordance with the effect of DCA observed on parasite proliferation, both DCA treatments (DCA1 and DCA2, as described in the legend of Fig. 2A) induced changes in cell division, leading to the formation of large atypical masses of damaged parasites seen as larger nondividing nuclei (Fig. 5A, white numbers). MitoTracker labeling showed smaller mitochondria, which may have originated from the fragmentation of the organelle in the process of division (Fig. 5A, white arrows). We also detected in some parasites alteration of apicoplast division, as observed by the presence of fragments with different sizes from this organelle (Fig. 5A, white asterisks). In sum, different DCA treatments induced the formation of masses with tethered parasites, collapsed or fragmented mitochondria, and irregular pieces of apicoplasts. We also measured the effect size of DCA on both organelles separately (Fig. 5B and C) based on the calculation of risk difference (RD), which measures the absolute difference in outcomes between the control group and the group receiving treatment (37). We detected a higher RD in the fragmentation of mitochondria (RD ≈ 0.68) than in the fragmentation of the apicoplasts (RD ≈ 0.15), although both effects were considered significant (*P* < 0.0001) (Table S2). These results indicate that DCA primarily modified the mitochondrial morphology as well as compromised the nuclear and, in some cells, the apicoplast division during endodyogeny.

10.1128/mSphere.01002-20.8 TABLE S2 Statistical tests on the numbers of altered mitochondrion and apicoplast organelles performed to generate Fig. 5. Download Table S2, XLSX file, 0.04 MB. Copyright © 2021 Ferrarini et al. 2021 Ferrarini et al. This content is distributed under the terms of the Creative Commons Attribution 4.0 International license.

10.1128/mSphere.01002-20.9 TABLE S3 Protein-coding genes detected as possible homologs to PDKs and BCKDS in human from apicomplexa and kinetoplastida (only one hit and best high-scoring segment pairs [hsp] per query). Download Table S3, XLSX file, 0.02 MB. Copyright © 2021 Ferrarini et al. 2021 Ferrarini et al. This content is distributed under the terms of the Creative Commons Attribution 4.0 International license.

### PDK and BCKDK are present in the genome of T. gondii.

It is well described that human PDK regulates PDH activity and is the target of DCA in tumor cells. In T. gondii, both PDH and BCKDH were described as able to convert pyruvate into AcCoA, but the kinases responsible for the regulation of their activities remain unknown. In order to get further insights into potential targets of DCA, we decided to investigate the conservation of these kinases in a comparison with the already-described targets of DCA in human. For this, we performed a whole-genome analysis of all possible PDK/BCKDK orthologs in T. gondii and we also searched for orthologs in other protozoan species to unravel the evolutionary history of these kinases in parasitic protists. In total, we detected putative orthologs for human PDKs/BCKDK in 12 species from the phylum Apicomplexa and 23 from the class Kinetoplastida from the phylum Euglenozoa. Four sequences from T. gondii were selected for further investigation. Overall, 130 sequences from other species were also included in the analyses, and these are reported in Table S3. All sequences from the initial analysis can be found in Table S4.

10.1128/mSphere.01002-20.10 TABLE S4 Complete BLAST results of homolog proteins of PDKs and BCKDK from human (all hits and hsp’s per query). Download Table S4, XLSX file, 0.1 MB. Copyright © 2021 Ferrarini et al. 2021 Ferrarini et al. This content is distributed under the terms of the Creative Commons Attribution 4.0 International license.

**FIG 6:**
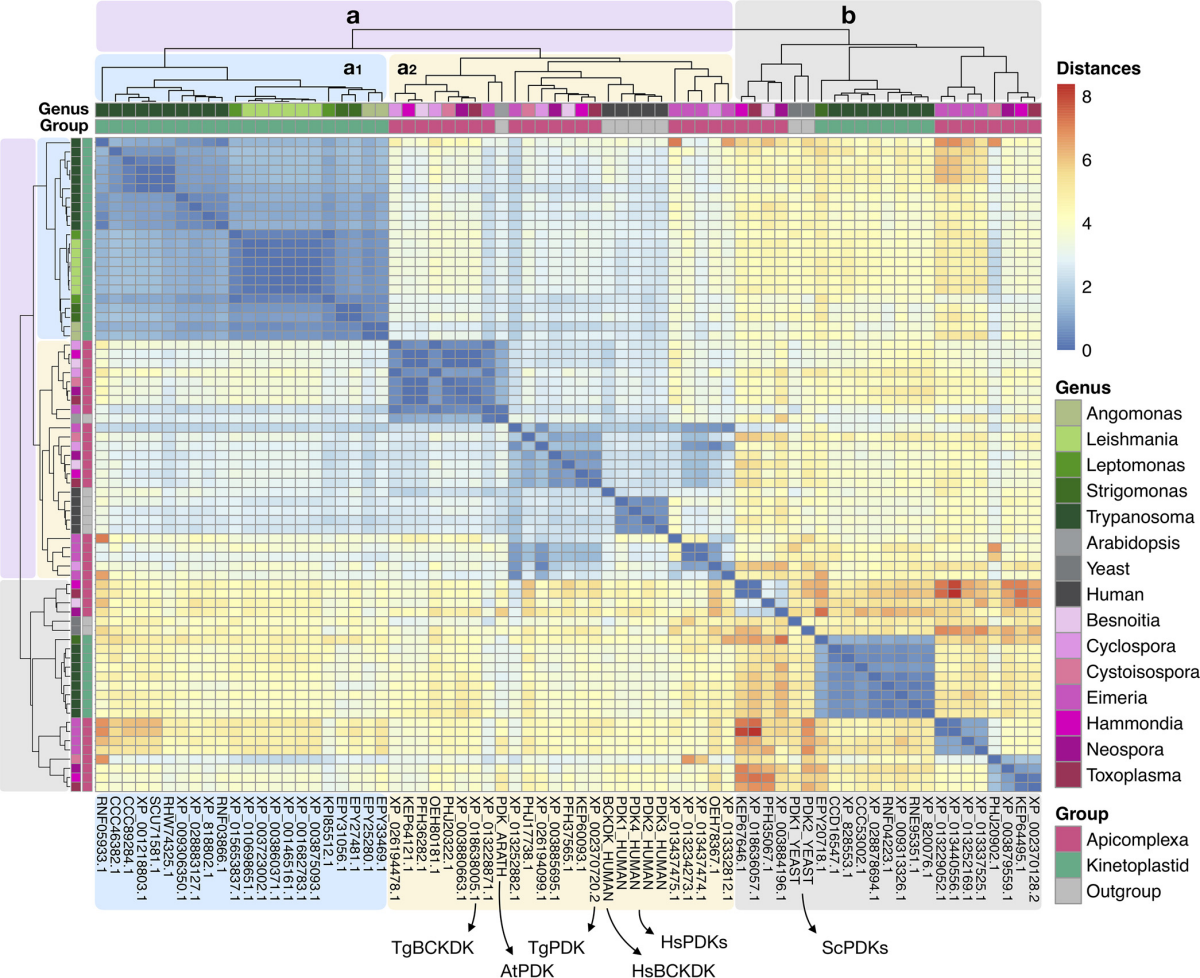
Identification of putative PDKs and BCKDKs in the T. gondii genome. Clustering analysis of 63 putative orthologs of HsPDKs from parasitic protists. Heatmap representing the pairwise distance scores computed with the bootstrap method (*n* = 1,000), the Poisson model for amino acid substitution, and the gamma distribution using multiple alignment with MEGA 7. On the top of the heatmap, the group apicomplexa (pink), kinetoplastida (green), or potential outgroups (gray) as well as the genera within each group are given. The scores of the distances are depicted as a gradient from blue (closer proteins) to red (more divergent proteins). Overall, two dichotomic groups, a and b, were detected. Interestingly, group a was further clustered into a1 with kinetoplastids and a2 with apicomplexans. Group a2 contained the proposed TgPDK and TgBCKDK, along with all 5 human kinases and PDK from *A. thaliana*.

Based on the clustering results and since the catalytic subunits of protein kinases were generally conserved, we kept only one representative organism for each species. Finally, we performed a clustering based on pairwise distances (Fig. 6), and we created a phylogenetic tree (Fig. S2) with a total of 63 selected proteins. Four PDKs and one BCKDK from human and two PDKs from Saccharomyces cerevisiae and one from Arabidopsis thaliana were used as potential outgroups. The clustering depicted a dichotomic structure, with two distinct populations (a in purple and b in gray in Fig. 6); however, proteins from group a were further subdivided into 2 subgroups (1 in blue and 2 in yellow), which separated quite well apicomplexans (depicted in pink shades) from kinetoplastids (depicted in green shades). The apicomplexan proteins generally clustered together with the outgroup proteins, whereas kinetoplastid proteins were more conserved within genera and divergent from the rest. Interestingly, group a1 recovered quite well the species phylogenetic tree of kinetoplastid species; group a2 on the other hand was much more disordered and mixed with the outgroups *Arabidopsis* and human. Proteins from group b were much more divergent than group a (given the high values of distance within this group) and seemed to be more similar to PDK from yeast.

10.1128/mSphere.01002-20.2 FIG S2 Molecular phylogenetic analysis by the maximum likelihood method for all putative PDK and BCKDK homologs in protozoan parasites. From 71 selected amino acid sequences (63 apicomplexan/kinetoplastida plus PDKs and BCKDKs from human, yeast, and *A. thaliana*, the evolutionary history was inferred by using the maximum likelihood method based on the Jones-Taylor-Thornton [JTT] matrix-based model). The tree with the highest log likelihood (−1,456.20) is shown. Initial trees for the heuristic search were obtained automatically by applying the neighbor-joining and BioNJ algorithms to a matrix of pairwise distances estimated using a JTT model and then selecting the topology with the superior log-likelihood value. A discrete gamma distribution was used to model evolutionary rate differences among sites (5 categories [+G, parameter = 1.2691]). The tree is drawn to scale, with branch lengths measured as the number of substitutions per site. All positions containing gaps and missing data were eliminated. There was a total of 16 positions in the final data set. Evolutionary analyses were conducted in MEGA7. Download FIG S2, PDF file, 0.1 MB. Copyright © 2021 Ferrarini et al. 2021 Ferrarini et al. This content is distributed under the terms of the Creative Commons Attribution 4.0 International license.

We could distinguish two kinases from T. gondii within group a2, closer to all four isoforms of Homo sapiens PDKs (HsPDK1, HsPDK2, HsPDK3, and HsPDK4), the experimentally validated targets of DCA. Originally, one of the T. gondii kinases (TGME49_219682) was annotated as “pyruvate dehydrogenase kinase,” but the second (TGME49_318560) was annotated as an “ATPase/histidine kinase/DNA gyrase B/HSP90 domain-containing protein.” In order to gain more information on these two proteins, we first aligned them directly with HsPDK1 and HsBCKDK (Fig. S3) to reconstruct an evolutionary tree. Even though they seemed to have diverged early in evolution, we could detect TGME49_318560 as more similar to HsBCKDK, whereas TGME49_219682 was phylogenetically closer to HsPDK1 (Fig. S4).

10.1128/mSphere.01002-20.3 FIG S3 Multiple alignment of selected PDKs and BCKDKs. We aligned the protein sequences of TgPDK and TgBCKDK with the four PDKs from human and BCKDKs from human and rat. We also used information from 3D PDB structures of human PDK1 and murine BCKDK. The green background indicates the signal peptides predicted for both TgPDK and TgBCKDK. The red background indicates amino acid sequence identity, and a yellow background and bold letters indicate sequence similarity. Download FIG S3, PDF file, 0.1 MB. Copyright © 2021 Ferrarini et al. 2021 Ferrarini et al. This content is distributed under the terms of the Creative Commons Attribution 4.0 International license.

10.1128/mSphere.01002-20.4 FIG S4 Evolutionary history of PDK and BCKDK from T. gondii. We compared directly the protein sequences of TgPDK and TgBCKDK with PDK1 from human and BCKDK from human and rat. Even though humans and protozoa diverged early in evolution, the duplication of the ancestral protein into PDK and BCKDK seems to be an event prior to the speciation of the two proteins. The evolutionary history was inferred by using the maximum likelihood method and JTT matrix-based model. The tree with the highest log likelihood (−5,439.98) is shown. Initial trees for the heuristic search were obtained automatically by applying the neighbor-joining and BioNJ algorithms to a matrix of pairwise distances estimated using a JTT model and then selecting the topology with the superior log likelihood value. The tree is drawn to scale, with branch lengths measured in the number of substitutions per site. This analysis involved 5 amino acid sequences. There was a total of 794 positions in the final data set. Evolutionary analyses were conducted in MEGA 7. Download FIG S4, PDF file, 0.04 MB. Copyright © 2021 Ferrarini et al. 2021 Ferrarini et al. This content is distributed under the terms of the Creative Commons Attribution 4.0 International license.

### TgPDK and TgBCKDK have conserved residues essential for PDK activity in humans.

**FIG 7:**
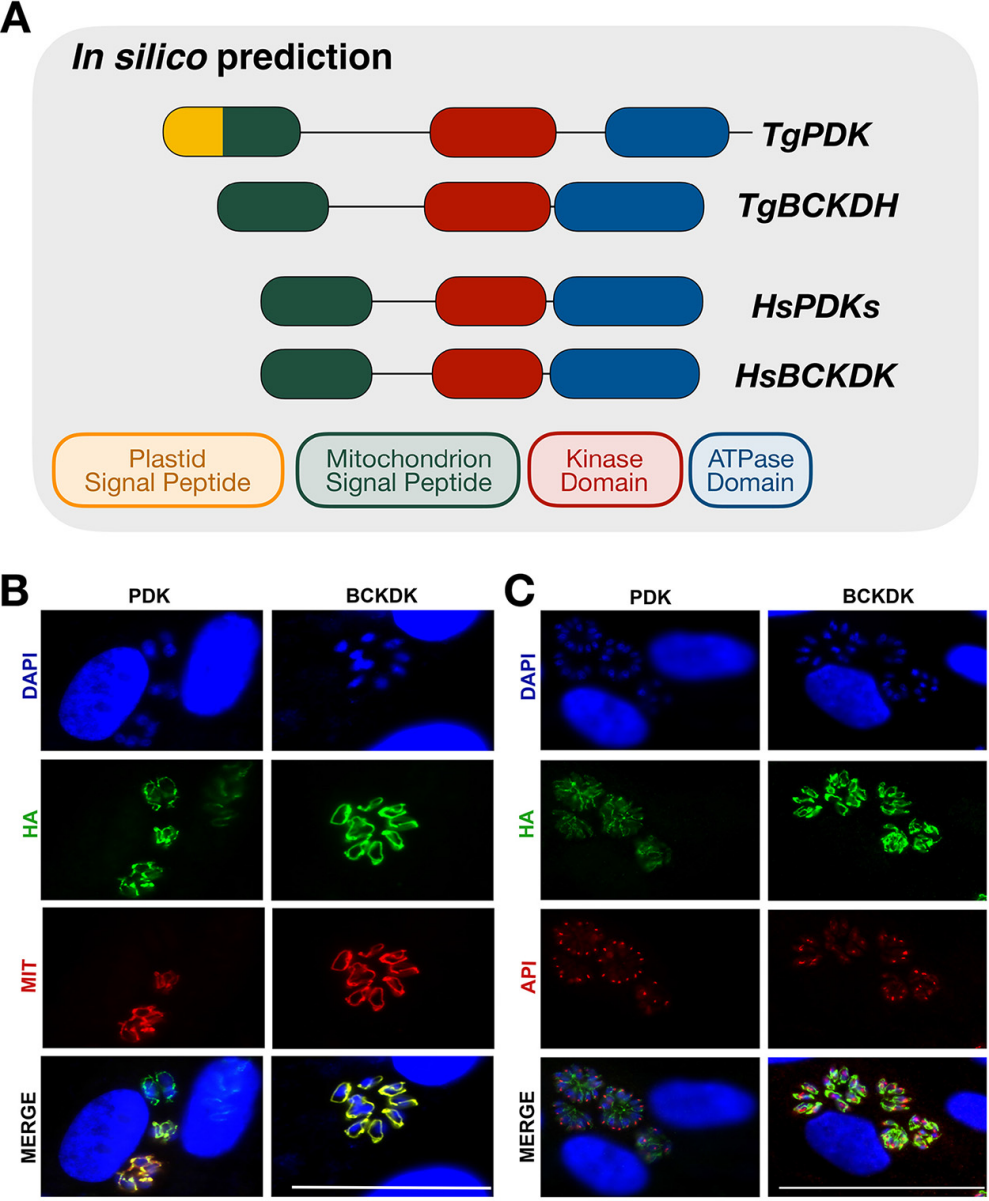
Domain conservation and subcellular localization of the proteins TgPDK and TgBCKDK in T. gondii. (A) By comparing the conserved domains of HsPDK and HsBCKDK, we could detect that, overall, TgBCKDK seems to be more conserved than TgPDK. TgBCKDK has a strong predicted mitochondrion signal peptide (in green), whereas TgPDK presents both a mitochondrion signal peptide and an apicoplast signal peptide (in yellow). The kinase domain (red) is slightly larger in T. gondii kinases than in the human kinases, and the ATPase domain (blue) was predicted to have the same size for all kinases analyzed here. The cellular localization of TgPDK and TgBCKDK (HA tag, in green) was determined with the colocalization of mitochondrial protein HSP60-RFP (B) and apicoplast protein CPN60 (C), both of which stained red; nuclei are marked with DAPI (blue). Bar, 50 μM.

We proceeded to further investigate the active sites of the two kinases and the interaction residues with the dehydrogenases in order to predict whether both could be proposed as targets of DCA. For this, we aligned both kinases from T. gondii with the PDKs and BCKDK from human as well as BCKDK from rat and searched for several important residues for PDK activity, PDK-DCA interaction, and PDH-PDK interaction as previously reported (38–41). A summary of the conservation of 30 residues is shown in Table 1. The two proteins had around 50% identity in their residues (in green, 14/30 for TgPDK and 16/30 for TgBCKDK), as well as conservative replacement of 15% of amino acids (in yellow, 5/30 for both proteins). However, TgBCKDK had a general structure of domains more similar to those in human PDKs than to those in TgPDK (Fig. 7A). From signal peptide analyses, we were able to detect a strong mitochondrial signal in TgBCKDK, whereas TgPDK had both plastid and mitochondrial signals predicted (Table 1; Fig. 7A). In this way, it is possible that both proteins are functional (given the conservation of important residues) and may potentially target pyruvate dehydrogenases in T. gondii.

**TABLE 1:**
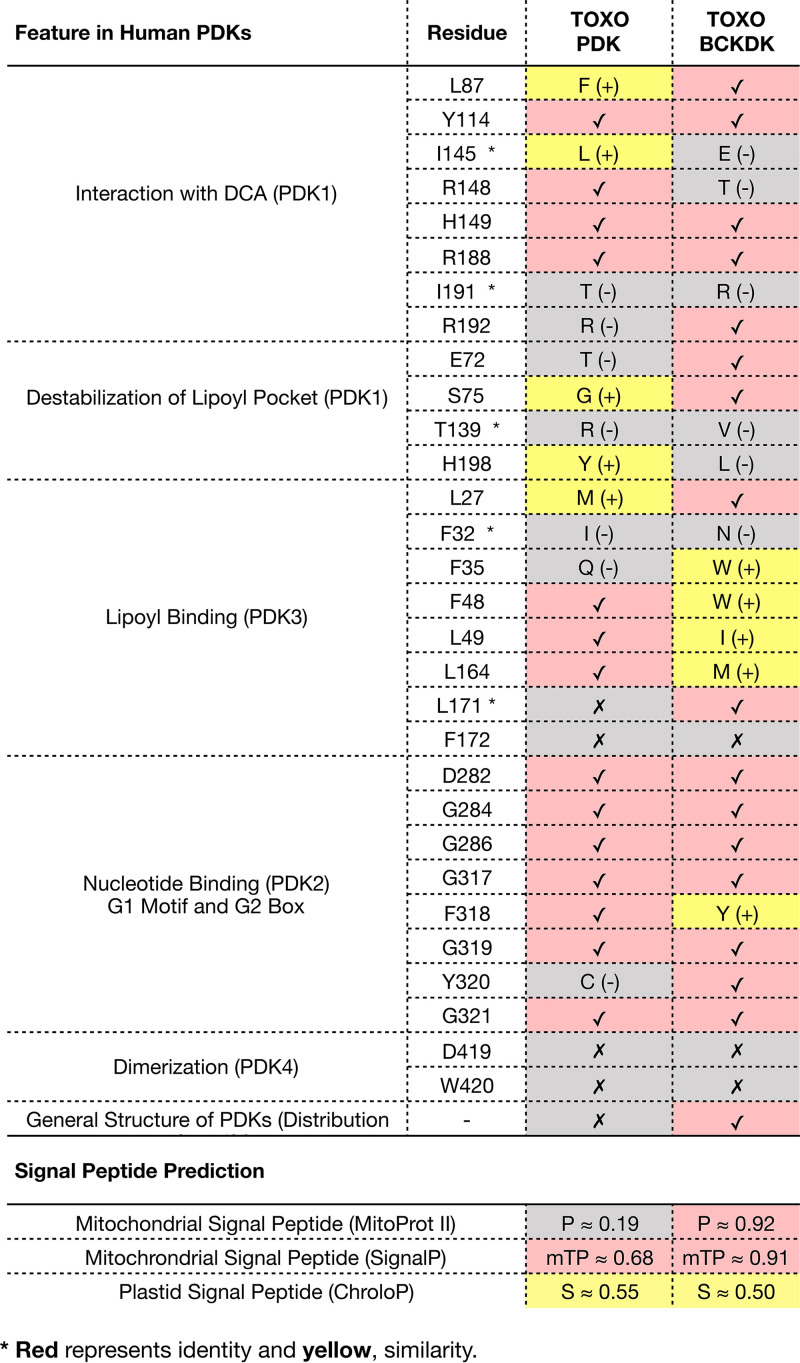
Important residues for PDK activity in human in comparison with those of TgPDK and TgBCKDK^*a*^

a P, mitochondrial signal peptide; mTP, MitoTracker SignalP; S, plastid signal peptide.

### TgPDK and TgBCKDK are localized in the mitochondrion.

To further assess whether TgPDK and TgBCKDK were present only in the mitochondrion or possibly in other organelles, such as apicoplasts, we decided to experimentally verify their subcellular localization. To localize both kinases, we tagged the proteins with the hemagglutinin (HA) epitope, while the mitochondria and apicoplasts were localized by the detection of target proteins, as described in Materials and Methods. Confirmation of genomic insertion is provided in Fig. S5. TgPDK or TgBCKDK was stained in green, while mitochondria (Fig. 7B) or apicoplasts (Fig. 7C) were stained in red. The merged images demonstrated that the two kinases colocalize with mitochondrial proteins. Neither protein colocalized with apicoplasts. This result confirmed that both proteins were exclusively located to the mitochondrion and thus may potentially regulate the activity of the main protein responsible for the pyruvate dehydrogenase activity (TgBCKDH) in this organelle.

10.1128/mSphere.01002-20.5 FIG S5 HA tagging of TgPDK and TgBCKDK. (A) Scheme of the genomic insertion strategy using the plasmid pLIC.HA.HXGPRT in the RH Δ*hxgprt* Δ*ku80* strain. (B) Confirmation of genomic insertion using PCR. This figure is comprised of 3 different electrophoresis gels as denoted by the black lines. For the Tg*PDK* gene, the primer pair 3 and 4 amplified a fragment of 1,861 bp (lane 1), and the primer pair 1 and 4 amplified a 1,445-bp fragment (lane 2). For the Tg*BCKDK* gene, the primer pair 3 and 4 amplified a 2,020-bp fragment (lane 5), and the primer pair 1 and 4 amplified a 1,649-bp fragment (lane 6). The negative control (Control) for each PCR was performed without DNA (lanes 3, 4, 7, and 8). (C) Western blot analysis for the detection of HA-tagged proteins in transfected parasites using rat anti-HA antibody. Nontransfected parasites were used as a negative control. The primers used in this PCR assay were as follows: 5′-TACTTCCAATCCAATTTAATGCCCGACGAAACTCCGTCTC-3′ (1-PDK), 5′-AACGAAGCTGGCGGACTC-3′ (3-PDK), 5′-TACTTCCAATCCAATTTAATGCTAGATTTCGATTAATATTCTCTGTGG-3′ (1-BCKDK), 5′-GCGCAGGAGAAGAATGCATG-3′ (3-BCKDK), and 5′-GCATAATCGGGCACATCATAG-3′ (4-HA). Download FIG S5, PDF file, 0.2 MB. Copyright © 2021 Ferrarini et al. 2021 Ferrarini et al. This content is distributed under the terms of the Creative Commons Attribution 4.0 International license.

## DISCUSSION

Analogously to the Warburg effect, glycolysis has been a strategy of *Plasmodium* for rapid proliferation, and the erythrocytic stages of this parasite have evolved toward aerobic fermentative glycolysis instead of mitochondrial oxidative phosphorylation (42). However, the reasons for this dependence on glycolytic fermentation remain unclear (43). Remarkably, the mitochondrial metabolism in apicomplexan organisms is different from that of other species, since the key enzyme of the complex for generation of energy (PDH) is absent in the mitochondrion and is present in the apicoplast (44). In most eukaryotes, the branched-chain α-keto acid dehydrogenase (BCKDH) is involved in the degradation of BCAA, but it was described to take over the PDH function in the mitochondrion of T. gondii, generating AcCoA from pyruvate (35). Usually, PDH activity is regulated by PDK (45); however, the regulatory function of this enzyme over PDH activity remains unknown in apicomplexan parasites.

The mitochondrial metabolism is required for parasite survival and is tightly related to the apicoplast metabolism, a specific and essential organelle from Apicomplexa (46–48). Thus, both organelles contain metabolic pathways and provide interesting candidates for drug targeting. DCA was first described in 2007 as a promising selective anticancer agent and as a generic drug that can be administered orally (49). It is known that PDK is the target of DCA in mammals (19), and this drug also has been used for complementary treatment of malaria; nevertheless, the mode of action of this compound on apicomplexan metabolism and morphology has never been investigated.

We assumed that DCA treatment would be useful for investigating regulatory factors of PDH activity, in addition to unraveling new potential targets of drug therapy. To this purpose, we first analyzed the toxic effects of DCA on NHDF in order to show that no secondary effects were detected and that they were suitable for the parasite infection experiments (Fig. 1). Using the OECD guidelines, we were able to classify DCA as in category 5 of drugs, which includes compounds without toxicity effects (33). Additional analyses (proliferation, apoptosis, and necrosis) demonstrated that DCA had no toxic effects on NHDF used in the infection experimental assays. This was not surprising since DCA had already been described as not toxic to normal cells (19).

Based on the calculated IC_50_, we chose the optimal concentration of 16 mM for our experiments to ensure a maximum effect on the parasites without causing any damage to the host cells. We demonstrated that 16 mM DCA was toxic to the parasite by impairing parasite growth and infection, possibly affecting both invasion and proliferation (Fig. 2 and 3). Nevertheless, more experiments should be carried out in order to verify this assumption. Indeed, DCA led to the formation of large masses of damaged parasites, incapable of originating normal daughter cells. However, lethal effects for parasites were not highly evident with the lower concentrations tested (Fig. S6). These results are in agreement with the literature that describes DCA to be effective only in millimolar ranges of concentration for *in vitro* effects of human cells (50–52). In those papers, DCA has been proposed to be used in synergy with other compounds as an alternative to reduce the drug concentration and to increase the benefit of DCA treatment.

10.1128/mSphere.01002-20.6 FIG S6 Plaque assay to detect the effects of different concentrations of DCA on T. gondii. (A) Effects of treatments of DCA in 2 concentrations (16 mM and 7 mM). Control 1, no DCA pretreatment of NHDF plus RH tachyzoites; control 2, NHDF pretreated with DCA for 48 h plus RH tachyzoites; DCA 1, NHDF pretreated with DCA for 48 h plus RH tachyzoites plus 16 mM DCA; DCA 2, no DCA pretreatment plus RH tachyzoites plus 16 mM DCA; DCA 3, no DCA pretreatment plus RH tachyzoites plus 16 mM DCA after 2 h postinvasion. Significance was tested with a Wilcox test (***, *P* < 0.001). (B) Plaque assay showing that even though the numbers of plaques were significantly reduced between the controls and the two concentrations of DCA tested, the use of 16 mM decreased also the sizes of the plaques, which did not happen with 7 mM DCA. Download FIG S6, PDF file, 2.3 MB. Copyright © 2021 Ferrarini et al. 2021 Ferrarini et al. This content is distributed under the terms of the Creative Commons Attribution 4.0 International license.

PDK is overexpressed in tumors, causing the Warburg effect and consequently leading to an antiapoptotic and proliferative environment. DCA inhibits PDK and reverts the suppression of mitochondrial metabolism, inducing apoptosis and an antiproliferative environment, causing impairment of a tumor’s growth both *in vitro* and *in vivo* (19, 24, 53).

We thus performed metabolic assays in order to investigate how DCA may affect this parasite and observed that the treatment of T. gondii with DCA stimulated PDH activity (Fig. 4A). These results suggest that the PDH activity in T. gondii might also be regulated by PDK, as previously reported in cancer cells (19) and, consequently, that PDK may be a target of DCA in these parasites. Furthermore, we observed that DCA reduced the Ca^2+^ cytoplasmic levels (Fig. 4E). Mitochondrial Ca^2+^ uptake in vertebrate cells is important for regulating the activities of three mitochondrial dehydrogenases: PDH, 2-oxoglutarate, and isocitrate dehydrogenases. Intramitochondrial Ca^2+^ stimulates all three dehydrogenase activities, leading to an increase in energy metabolism (54–56). It is worth observing that, until now, no Ca^2+^-regulated dehydrogenases have been reported in apicomplexan parasites (57). To our knowledge, this is the first report of the regulation of PDH activity associated with changes in Ca^2+^ levels in apicomplexans.

In our analysis, the increase in PDH activity was followed by an increase of ATP levels but not by reactive oxygen species (ROS) production (Fig. 4D and F). Intriguingly, it has already been described that DCA increases ROS and ATP due to activation of oxidative phosphorylation (58) and that DCA in synergism with metformin also potentiates ROS production in breast cancer cells (59). Thus, our analysis indicates slight differences in DCA effects in parasites, since we observed ATP increasing without differences in ROS production. However, irrespective of the ROS levels, our results demonstrate that the DCA treatment caused a global unbalanced mitochondrial activity. This was probably due to an activation (or lack of repression) of TgBCKDH, since this is the enzyme responsible for the major PDH activity in this parasite (35). However, we cannot exclude the possibility that TgPDH in the apicoplast was also affected by the treatment.

To better understand the cellular effects of DCA on both apicoplast and mitochondrion, we decided to investigate morphological alterations of those organelles by superresolution microscopy (Fig. 5). Indeed, the treatment with DCA modified the mitochondrion morphology. Smaller circular mitochondria were observed in parasites that were unable to properly undergo the endodyogeny process. Although those smaller mitochondria (or pieces originating from it) seemed to have normal membrane potential, as evidenced by the MitoTracker probe, we can speculate that it might be a consequence of unbalanced mitochondrial activity caused by DCA. The treatment with DCA also compromised the apicoplast division in a few cells, generating organelles with irregular sizes. Indeed, several metabolites produced in the mitochondrion are necessary for apicoplast metabolism (47). Moreover, since TgPDH is localized in the plastid (34) and pyruvate is a key metabolic precursor for the synthesis of fatty acids shorter than C_18_ (FAS2), FAS2 alteration may also compromise the synthesis of long-chain fatty acids, which occurs in cooperation with the endoplasmic reticulum (60). This may jeopardize the correct input of membrane supply for daughter cell formation and subsequent parasite proliferation. We cannot exclude the possibility that this is a consequence of metabolic alterations caused directly or indirectly by DCA; however, further investigations are necessary to unravel if those morphological events were indeed the cause or the consequence of parasite death.

In order to get some evidence of whether the observed metabolic alterations were triggered by PDK inhibition, we decided to analyze the genes coding for PDK in the genome of T. gondii. At first, we had four potential candidate orthologs. We then performed a general screening of this protein family in all parasitic protists to understand its evolutionary history (Fig. 6). Through sequence similarity and phylogenetic analyses, we were able to narrow down numbers to two proteins that clustered together with both HsBCKDK and HsPDK. In mammals, BCKDK inhibits the function of BCKDH present in mitochondria and decreases the catabolization of the corresponding keto acids (61, 62), but similarly to PDK, BCKDK has never been annotated or studied in apicomplexans.

The phylogenetic analysis alone was not enough for us to determine whether or not both enzymes in T. gondii are direct targets of DCA. We therefore searched for conserved signals and regulatory domains that would give us indications about the localization and activities of these proteins. Indeed, the protein that we named TgPDK seemed to be evolutionarily closer to isoform HsPDK1 (Fig. S4). Nevertheless, based on Fig. 7A, we could detect that the general structure of TgBCKDK was closer to those of human PDKs. Regarding their putative location, TgBCKDK had a strong signal for mitochondrial localization, whereas TgPDK had both a mitochondrion and an apicoplast peptide signal. In mammals, both PDK and BCKDK are found in the mitochondrion (63, 64), and two proteomics studies showed the presence of these two proteins enriched in mitochondrial fractions in T. gondii (65, 66). We further validated the mitochondrial subcellular localization of both TgPDK and TgBCKDK by immunofluorescence assays (Fig. 7B and C).

Although further steps in the characterization of such proteins are essential to understand these pathways in T. gondii, this work has clearly demonstrated that DCA directly impairs the proliferation and infection of this parasite without affecting the human cells. We also demonstrated that this drug increased the PDH activity in this parasite, and we further propose that DCA can effectively target either one (or both) of the kinases that are indeed located in the mitochondrion. Further analyses of the structure of TgPDK and TgBCKDK will help to identify their functional roles and specific features for inhibitor design since lineage-specific variations of the structures of kinases in *Apicomplexa* can also be used for the design of selective kinase inhibitors (67).

Indeed, we demonstrated that DCA treatment was lethal to T. gondii without causing any collateral effect to the host cells. We are aware that the concentration of DCA used here to repress the parasite infection was higher than the usual concentration expected for effective drugs. However, we confirmed that DCA increased PDH activity and led to unbalanced mitochondrial activity as well as morphological alterations of both mitochondrial and apicoplast organelles in tachyzoites. Furthermore, we confirmed the presence in the genome of putative kinases that may regulate the PDH activity, verified their mitochondrial subcellular localization, and provided *in silico* data to assign them as potential binding enzymes of DCA. For this reason, we believe that the DCA treatment in addition to the functional characterization of these kinases will provide interesting insights into the regulation of pyruvate metabolism as well as the discovery of potential targets to be used to fight toxoplasmosis in the future.

## MATERIALS AND METHODS

### DCA cytotoxicity of human fibroblast cells.

Normal human dermal fibroblasts (NHDF) (Lonza, Basel, Switzerland) were cultivated using Dulbecco’s modified Eagle’s medium (DMEM) supplemented with 10% of fetal bovine serum (FBS), 2 mM l-glutamine, 100 U/ml of penicillin, and 100 μg/ml of streptomycin. The cells were maintained at 37°C in a 5% CO_2_ atmosphere. NHDF were used in all experiments until the 13th passage. The DCA was purchased from Sigma-Aldrich (St. Louis, MO, USA), solubilized, and diluted in culture medium immediately before use. To verify the DCA cytotoxicity, the NHDF were submitted to the neutral red uptake (NRU) assay, based on ICCVAM and OECD 129 guidelines (30, 68, 69). Sodium dodecyl sulfate (SDS) was used as the control drug, as recommended by ICCVAM, and a negative control (medium only) was included in all the assays. The first step consists in the plating of the cells on 96-well plates, with 3.5 × 10^3^ cells/well. After 24 h, at 37°C with 5% CO_2_, a high-concentration solution of DCA was prepared, diluted serially eight times, and then added to the cells. After 48 h, the cells were rinsed with warmed Dulbecco’s phosphate-buffered saline (D-PBS) and incubated with 25 μg/ml of the supravital dye neutral red solution (Sigma-Aldrich, St. Louis, MO, USA) for 3 h at 37°C. Afterwards, the neutral red withheld by viable/living cells was extracted with a solution composed of 50% ethanol and 1% acetic acid (Merck, NJ, USA). Plates were then shaken for 20 to 45 min, and the optical densities of the samples were measured at 540 nm on a Synergy H1 multimode reader (Biotek, VT, USA). In all assays, a separate plate of SDS concentrations was used as a positive control. The data obtained were analyzed according to the ICCVAM recommendations, and the IC_50_ values and LD_50_ predictions were accomplished (30, 32, 70–73). Data were converted into a percentage of the control values. In order to calculate IC_50_ values, data were transferred to GraphPad Prism (GraphPad Software Inc., San Diego, CA) to apply a sigmoidal dose-response variable slope with four parameters. The parameters were then used with the rearranged Hill function. Outliers were analyzed using the Grubbs test (available online at http://graphpad.com/quickcalcs/Grubbs1.cfm). The IC_50_ was also used to predict the LD_50_ and GHS class for test substances. The IC_50_ geometric mean was used to predict the LD_50_ by RC rat-only millimole regression using the formula log LD_50_ (mmol/kg) = 0.439 log IC_50_ (mM) + 0.621 (*R*^2^ = 0.452). After calculating the LD_50_, it was possible to predict the DCA toxicity class. Our assay was correct in the prediction of the GHS class of toxicity.

### Human fibroblast proliferation assays.

We used Ki67 as a marker, since this is a nuclear protein known to be expressed when cells are proliferating but which remains inactive when cells enter the G_0_ phase of the cell cycle (74–76). The NHDF (3.5 × 10^3^ cells per well) were cultivated for 48 h with or without DCA in 96-well plates and were fixed using a 4% paraformaldehyde solution. Subsequently, the cells were permeabilized in 0.5% Triton X-100 in PBS and blocked with 1% bovine serum albumin (BSA) in PBS. The cells were further incubated with rabbit-anti Ki67 primary antibody (Abcam, MA, USA) and detected using Alexa Fluor 488-conjugated goat anti-rabbit antibody (Invitrogen, CA, USA). Nuclear DNA was stained with 4′,6′-diamidino-2-phenylindole (DAPI). The images were obtained using the Leica DM6000 fluorescence microscope, and 60 pictures of each cultured condition per well were taken and analyzed using the ImageJ software, which quantifies the Ki67- and DAPI-positive stained cells.

### Cell cycle evaluation for human fibroblasts.

The NHDF (1 × 10^6^ per sample) were harvested for 48 h with and without 16 mM DCA, trypsinized, and fixed with 70% ice-cold ethanol at −20°C for 2 h. Next, the samples were washed once with PBS (1×; 700 g/5 min), incubated with 500 μl propidium iodide solution (3.4 mM Tris-HCl, pH 7.4; 0.1% [vol/vol] nonylphenoxy-polyethoxy ethanol [NP-40]; 700 U/liter RNase A; 10 mM NaCl; and 30 μg/ml of propidium iodide) for 30 min at room temperature, and protected from the light. After another wash, the samples were resuspended in PBS (1×) and analyzed on a BD FACSCanto II flow cytometer, with 1 × 10^4^ events recorded, at a low flow rate. The data analysis was performed on FlowJo software.

### Apoptosis and necrosis level measurement for human fibroblasts.

The NHDF were cultivated for 48 h without or with 16 mM DCA, and 5 × 10^5^ cells were incubated with the apoptosis detection probe annexin V and the necrosis marker 7-aminoactinomycin D (7-AAD), according to the manufacturer’s protocol (BD Pharmingen, NJ, USA). Samples were analyzed in a FACSAria II (BD Biosciences, NJ, USA) flow cytometer, which accounted for 1 × 10^4^ events. The positive marker for each probe was analyzed with the FlowJo 10 software, with a quadrant gate strategy used to separate the cells stained only with 7-AAD (necrotic cells), only with annexin V (early apoptotic cells), and with both probes (necrotic and/or late apoptotic cells).

### Statistical analysis for human fibroblast assays.

Both paired and unpaired Student *t* tests were performed (indicated in the legends of the graphics) using GraphPad Prism. Data are presented as bar plots of means ± standard errors from three independent experiments.

### Parasite culture.

Tachyzoites of the T. gondii RH strain (type I) and RH strain-acyl carrier protein (ACP, kindly provided by Erica Martins-Duarte)-yellow fluorescent protein (YFP) were maintained *in vitro* by passaging them in NHDF. They were grown in 75-cm^2^ tissue culture flasks containing DMEM supplemented with 10% FBS, 2 mM l-glutamine, 100 U/ml penicillin, and 100 μg/ml streptomycin at 37°C under a 5% CO_2_ atmosphere. To purify the tachyzoites, infected NHDF were lysed through a 27-gauge needle and the tachyzoites were filtered using a 5-μm-pore-size filter from Millipore (Burlington, MA, USA).

### Parasite DCA treatment.

For all the experiments, the cells and parasites were treated with 16 mM DCA diluted in DMEM supplemented with 10% FBS, 2 mM l-glutamine, 100 U/ml penicillin, and 100 μg/ml streptomycin. We performed the experiments under five different conditions: (i) the NHDF were infected with tachyzoites for 1 h and not treated with DCA (control 1); (ii) the NHDF were pretreated with DCA for 48 h, washed to remove the drug, and infected with tachyzoites for 1 h (control 2); (iii) the NHDF were pretreated for 48 h with DCA, infected with tachyzoites for 1 h, and kept with DCA in the medium until the end of the analysis (DCA 1); (iv) the NHDF were infected with tachyzoites for 1 h and kept with DCA in the medium until the end of the analysis (DCA 2); and (v) the NHDF were infected with tachyzoites for 1 h, and DCA was added to the medium only after 2 h of infection (DCA 3).

### Parasite growth analysis by plaque assay.

We performed a plaque assay based on previous work (77). NHDF were grown to confluence overnight in 6-well cell culture plates and were infected with 1,000 tachyzoites from the RH strain per well. The plates were incubated for 7 days at 37°C in a 5% CO_2_ environment. After this, the culture medium was removed and the infected NHDF were fixed with cold 100% methanol for 20 min at room temperature, stained with a Giemsa solution for 30 min at room temperature, and washed twice with PBS. The number of plaques (areas in the cell culture without cells) was determined in each well (77, 78). Images were taken using a Zeiss microscope. This experiment was performed in biological triplicates.

### Parasite infection assays.

The assays were performed with variations from previous works (79, 80). Confluent NHDF cultures were grown on glass coverslips, incubated for 1 h with 5 × 10^5^ tachyzoites, and washed twice with PBS to remove noninvading parasites. Further fresh medium without or with DCA was added. After 23 h, cultures were fixed with 4% formaldehyde in PBS for 20 min at room temperature. Cells were permeabilized with 0.2% Triton X-100 in PBS for 15 min, and nonspecific sites were blocked with PBS containing 3% bovine serum albumin and 0.2% Triton X-100 for 30 min before incubation with anti-IMC antibody, diluted 1:500, for 1 h at room temperature. Cells were incubated with the secondary antibody Alexa Fluor 488-conjugated anti-rabbit antibody, diluted to 1:1,000, for 1 h at room temperature. Slides were stained with DAPI to detect nucleic acids. Cells were washed with PBS several times and mounted onto slides. Image acquisition was done on a Zeiss fluorescence microscope, and the number of vacuoles per field and the number of parasites per vacuole were computed.

### Statistical analysis for parasite assays.

Statistical analyses were performed using GraphPad Prism. The data are means ± standard errors from three independent experiments.

### Evaluation of the PDH and BCKDH activities.

The protein extracts were obtained from 108 purified tachyzoites. After centrifugation at 8,000 × *g* for 15 min, the parasites were resuspended in 1 ml of lysis buffer (200 mM Tris-HCl, 1 mM EDTA, 250 mM sucrose, 100 mM NaCl, 0.1% Triton X-100, pH 7.9) containing protease inhibitors (10 μg/ml TLCK [*N*α-*p*-tosyl-l-lysine chloromethyl ketone], 40 μM E-64, 4 μg/ml aprotinin). The cells were lysed by sonication using the Vibra-Cell ultrasonic VCX 130 liquid processor, with 5 cycles of 30 s and 30-s rest intervals between the cycles, and centrifuged at 12,000 × *g*. The supernatant containing the protein extracts was used for enzyme activity assays. The protein concentration was quantified by the Bradford assay. The activities were performed with red-ox enzymes/complexes having NAD^+^ as a cofactor and NADH as a by-product. Their activities were measured by following up the NAD^+^ reduction to NADH by determining absorbance (340 nm, ε= 6, 200 M^–1^ cm^−1^) in a Synergy H1 multimode microplate reader (BioTek, USA). In both cases, reaction mixtures were prepared to contain 0.1 mM CoA-sodium salt hydrate (SH), 0.2 mM thiamine pyrophosphate (TPP), 2.5 mM NAD^+^, 3 mM MgCl_2_ at pH 7.2, and different concentrations of pyruvate (when the PDH activity was measured) or 4-methyl-2-oxovaleric acid (when the BCKDH activity was measured). The reaction mixtures were distributed in 96-well plates (100 μl per well), and the measurements were initiated by the addition of T. gondii cell extracts. The progression of the reactions was monitored at room temperature for the first 10 min.

### Bioenergetics assays.

First, the cells (2 × 10^7^ cells/ml) were incubated in PBS supplemented or not with 5 mM glucose, and each of these samples was treated or not treated with 16 mM DCA. The functionality of the mitochondrial dehydrogenases (usually considered a reliable indicator of mitochondrial viability) was evaluated by assessing the irreversible reduction of resazurin (7-hydroxy-3H-phenoxazine-3-one 10-oxide; Sigma) to resorufin (7-hydroxy-3H-phenoxazin-3-one), a red-ox fluorimetric indicator, used in viability assays for trypanosomatids and apicomplexans (81–85). Parasite samples (2 × 10^6^ cells/ml) from each one of the four groups described above were aliquoted in 96-well plates (100 μl per well) and incubated in the presence of 0.125 μg/μl resazurin for 3 h (in the absence of light) at 37°C. The fluorescence signal was measured in a Synergy H1 multimode microplate reader (BioTek, USA) at an excitation wavelength (*λ*_exc_) of 560 nm and an emission wavelength (*λ*_em_) of 590 nm.

### Evaluation of hydrogen peroxide production.

The evaluation of hydrogen peroxide production was made by using Amplex red as an indicator. As a positive control for an endogenous increase in peroxide production, we treated the parasites with 5 μg/ml oligomycin (Omy), which induces a hyper-polarization of the inner mitochondrial membrane and which favors the e-leakage increasing the intracellular content of peroxide. Parasites (2 × 10^6^ cells/ml) incubated with PBS supplemented or not with glucose and treated or not with DCA were aliquoted in 96-well plates (100 μl per well) and incubated in the presence of 10 μg/ml horseradish peroxidase and 2.5 μM Amplex red. Fluorescence was monitored at an *λ*_exc_ of 560 nm and an *λ*_em_ of 590 nm on a Synergy H1 multimode microplate reader (BioTek, USA). Calibration was performed using commercial hydrogen peroxide as a standard.

### Evaluation of intracellular ATP levels.

The intracellular concentration of ATP under each condition was determined by using a luciferase assay according to the manufacturer’s instructions (Sigma). ATP concentrations were estimated by using a calibration curve (ATP disodium salt; Sigma). Luminescence (*λ* at 570 nm) was detected using a Synergy H1 multimode microplate reader (BioTek, USA) as described previously (86).

### Analysis of intracellular Ca^2+^ levels.

Parasites for each condition were incubated with 5 *μ*M Fluo-4 AM (Invitrogen) for 1 h at 37°C. After this period, the cells were washed twice with Cytomix buffer (25 mM HEPES-KOH, 120 mM KCl, 0.15 mM CaCl_2_, 2 mM EDTA, 5 mM MgCl_2_, 10 mM K^+^-phosphate buffer, pH 7.2), resuspended in the same buffer at a cell density of 2 × 10^6^ cells/ml, and distributed in 96-well plates (100 μl per well) for fluorimetric readings. Readings were performed on a Synergy H1 multimode microplate reader (BioTek, USA) using a *λ*_exc_ of 490 nm and a *λ*_em_ of 518 nm.

### Statistical analysis for biochemical assays.

Statistical analyses were performed with one-way analysis of variance (ANOVA) and Tukey’s posttest with a significance (*P*) level of <0.05 in GraphPad Prism. The data are means ± standard errors from three independent experiments.

### Effect of DCA on mitochondria and apicoplasts.

In order to evaluate the effect of DCA on the mitochondria and apicoplasts of T. gondii parasites, tachyzoites expressing ACP-YFP and the fluorescent probe MitoTracker were used with NHDF under the following conditions. Untreated NHDF were infected with tachyzoites for 1 h (control 1). NHDF were DCA pretreated for 48 h, washed to remove the drug, and infected with tachyzoites (control 2). NHDF were pretreated for 48 h with DCA, washed, infected with tachyzoites for 1 h, and kept with DCA in the medium until the end of the analysis (DCA 1). NHDF were infected with tachyzoites for 1 h (DCA was added to the medium from the infection and kept until the end of the analysis) (DCA 2). The cells were washed with PBS and incubated with 50 nM MitoTracker red CMXRos (Invitrogen) for 45 min at 37°C in a 5% CO_2_ atmosphere. Soon after, they were washed and fixed with 4% formaldehyde for 30 min, and incubated with Hoechst 33342 at 0.5 μg/ml (ThermoFisher). Coverslips were mounted using Antifade Prolong Gold (Invitrogen). The analyses were performed with an Axio Observer microscope using the LSM 710 module with Plan-Apochromat 63×/1.4 DIC M27 Alpha objective oil (Zeiss, Germany). Super-resolution structured illumination microscopy (SR-SIM) images were acquired using the Zeiss Elyra PS.1 superstructure module (Jena, Germany). Images were processed using a manual structural lighting tool from ZEN Black (Zeiss, Germany).

### Statistical analysis for effect size of DCA on mitochondrion and apicoplast.

Quantification of DCA effect size was performed by assessing the number of altered mitochondria or apicoplasts in parasites per 100 cells. Statistical analyses were performed with a Fisher test between DCA-treated and control conditions, with at least two biological replicates in GraphPad Prism. The effect size of the drug was measured based on the methods described in reference 37, and we calculated the risk ratio, odds ratio, and risk difference between control and treatment groups.

### Phylogenetic analysis.

To determine if a pyruvate dehydrogenase kinase was present in the genome of T. gondii, we retrieved the protein sequences for the four human pyruvate kinases (HsPDK1, HsPDK2, HsPDK3, and HsPDK4) and the closely related BCKDK (HsBCKDK) and performed whole-genome analysis of possible orthologs in T. gondii. We also searched for orthologs in other parasitic protists (from the phylum Apicomplexa or the class Kinetoplastida from the phylum Euglenozoa) with available genomes to study the evolution of these kinases in these organisms. Reciprocal protein BLAST searches were performed with the NCBI-BLAST+ command line tool (87), and a hit protein was kept for further verification whenever the E value was <10^−5^ and the query coverage was >50%. After this, we (i) performed a multiple-sequence alignment using the neighbor-joining method, (ii) computed the pairwise distances of the sequences and built a maximum likelihood tree with the use of the MEGA 7 software (88) and MUSCLE algorithm (89), and (iii) performed a hierarchical clustering of sequences with Euclidean distances and visualized the results with the pheatmap package (90) from R.

### Comparative analysis of selected proteins.

To evaluate the active sites of TgPDK and TgBCKDK, we performed a multiple-sequence alignment of the selected sequences with the use of MEGA 7 and MUSCLE as previously explained. Known residues involved in the catalysis of PDK/BCKDK and interactions with DCA were analyzed in detail in order to infer if both kinases were predicted to be active and possibly targeted by DCA. The three-dimensional (3D) structures of human PDK1 (Protein Data Bank [PDB] accession no. 2Q8H) and murine BCKDK (PDB accession no. 1GJV) were also included in the analysis for secondary-structure information. We further evaluated if both dehydrogenase complexes were expected to be affected by PDK/BCKDK activity. All alignments were visualized with ESPript 3.0 (91). We also used the prediction softwares SignalP 5.0 (92), ChloroP (93), MitoProt II (94), and DeepLoc (95) to investigate the localization of both kinases.

### Subcellular localization of TgPDK and TgBCKDK by immunofluorescence assays.

One fragment of 1,000 bp corresponding to the C-terminal region of each gene product was amplified by PCR and cloned into the plasmid pLIC.HA.HXGPRT by ligation-independent cloning (LIC), as previously described (96). The sequences of oligonucleotides used were as follows: PDK F, TACTTCCAATCCAATTTAATGCCCGACGAAACTCCGTCTC; PDK R, TCCTCCACTTCCAATTTTAGCGTCCCGAGGCAGTCCG; BCKDK F, TACTTCCAATCCAATTTAATGCTAGATTTCGATTAATATTCTCTGTGG; and BCKDK R, TCCTCCACTTCCAATTTTAGCGCCTGGCGCTTTGTGTTC.

In order to insert the HA tag into the endogenous genes, the plasmid was linearized using SphI for the BCKDK plasmid and NcoI for the PDK plasmid before transfection into the RH Δ*hxgprt* Δ*ku80* strain and selection of transgenic parasites. The insertion of the HA tag was confirmed by PCR and Western blot analyses (Fig. S5). For immunofluorescence (IF) assays, intracellular parasites were fixed with 4% formaldehyde for 20 min at room temperature (RT), permeabilized with 0.2% Triton X-100 in PBS for 15 min, and blocked with 1% albumin serum and 0.2% Triton X-100 in PBS for 30 min at RT before incubation with anti-HA monoclonal antibody (Clone 3F10; Roche), diluted 1:500. Further, the antibodies were removed with PBS washes, and the samples were incubated with Alexa Fluor 488-conjugated anti-rat antibody diluted 1:800. For colocalization assays with apicoplasts, samples were incubated with anti-CPN60 antibody (97) diluted 1:1,000. This antibody was detected using Alexa Fluor 546-conjugated anti-rabbit antibody diluted 1:800. All antibodies were diluted in blocking solution and incubated for 1 h at room temperature. Cells were washed with PBS three times and mounted on slides with DAPI diluted 1:10,000 in PBS. For colocalization assays with mitochondria, the parasites were transiently transfected with the plasmid HSP60-RFP in pBTR (98) and the cells were processed as described above. Image acquisition was conducted using a Leica DMi8 microscope.
