# Supplementary figures and images for: Dichloroacetate and Pyruvate Metabolism: Pyruvate Dehydrogenase Kinases as Targets Worth Investigating for Effective Therapy of Toxoplasmosis
Source: mSphere. 2021 Jan 6;6(1):e01002-20. doi: 10.1128/mSphere.01002-20 (PMC7845590; doi:10.1128/mSphere.01002-20)

# BCKDH Activity

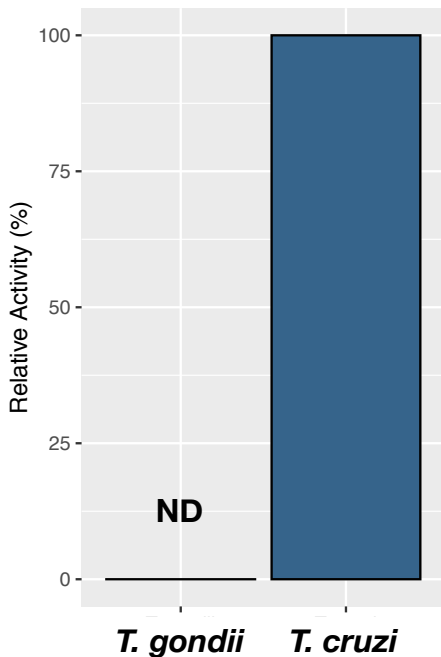

Supplement: FIG S1 [file mSphere.01002-20_sf001.pdf]

a

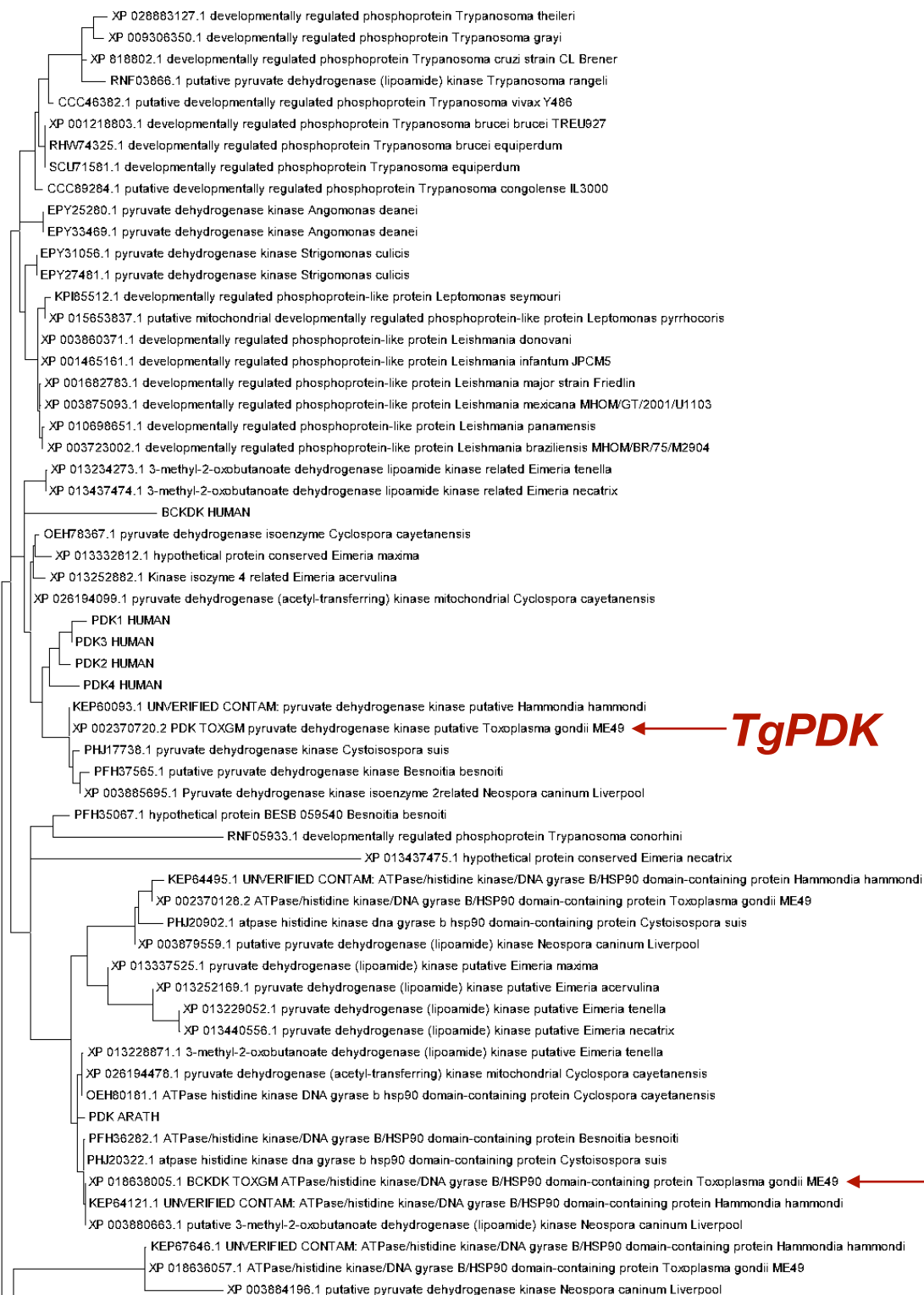

TgPDK

b

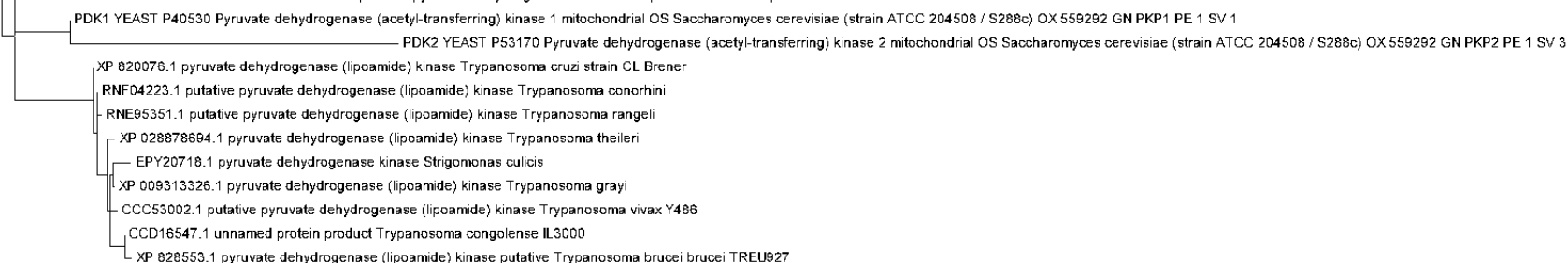

TgBCKDK

Supplement: FIG S2 [file mSphere.01002-20_sf002.pdf]

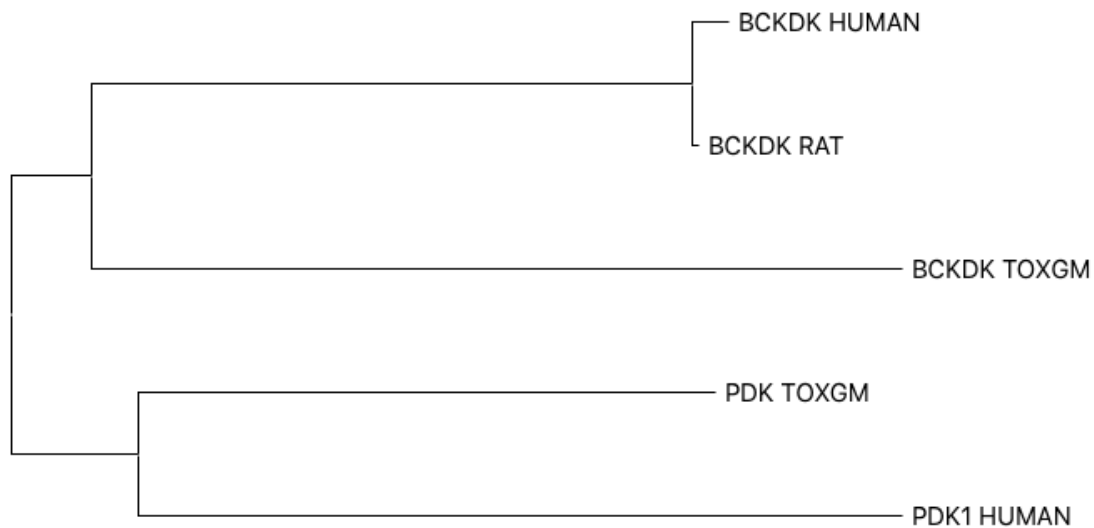

0.20

Supplement: FIG S4 [file mSphere.01002-20_sf004.pdf]

**A**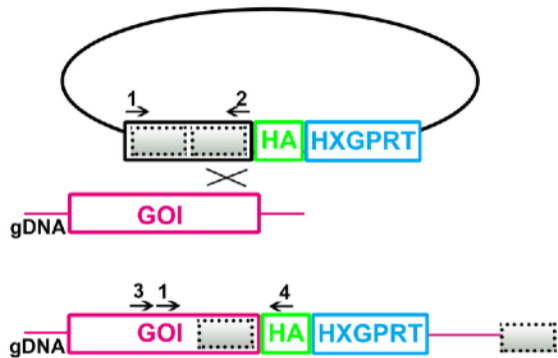**B**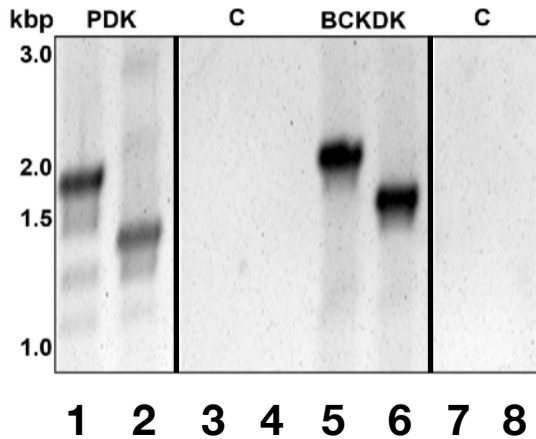**C**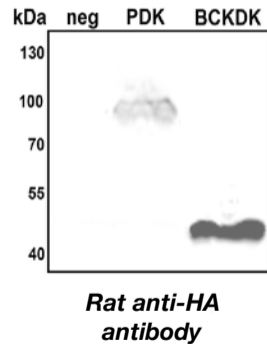

Supplement: FIG S5 [file mSphere.01002-20_sf005.pdf]

**A**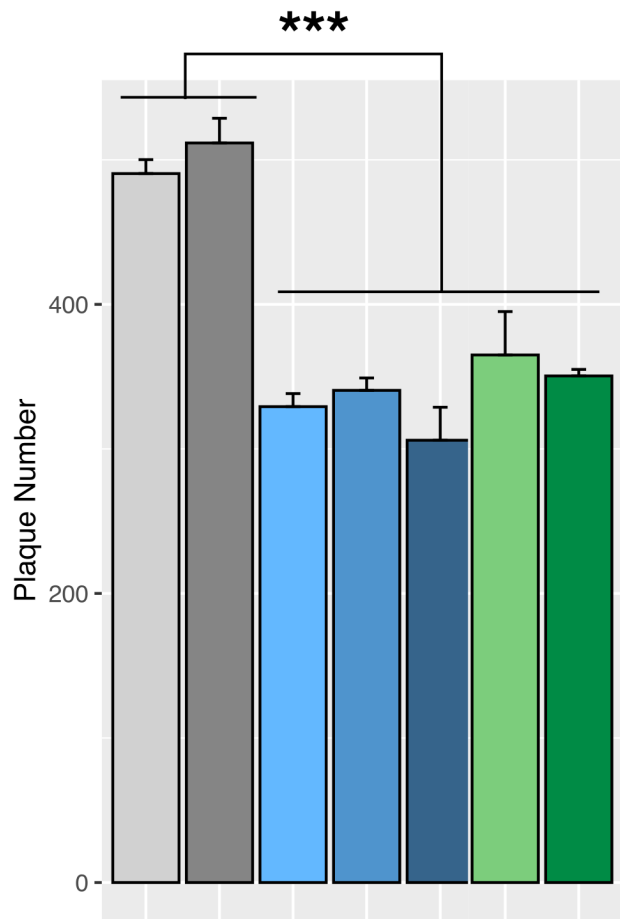**Type**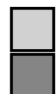

Control 1  
Control 2

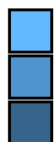

DCA (16mM) 1

DCA (16mM) 2

DCA (16mM) 3

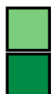

DCA (7 mM) 2

DCA (7 mM) 3

**B**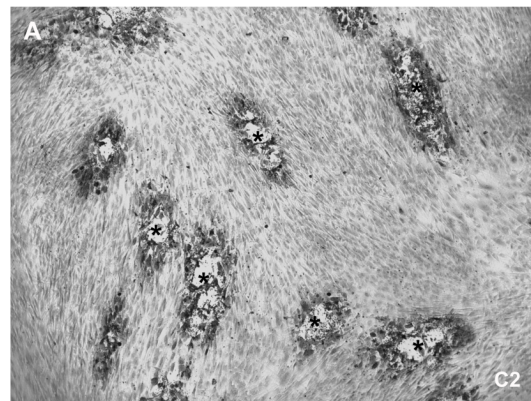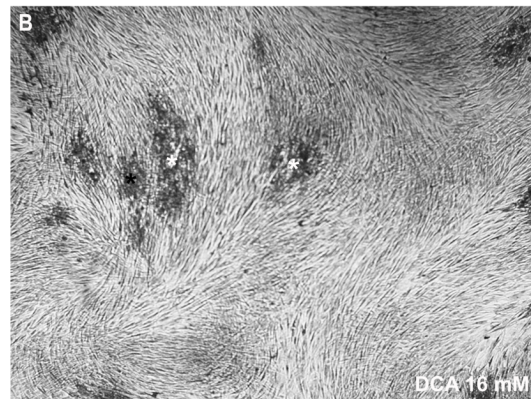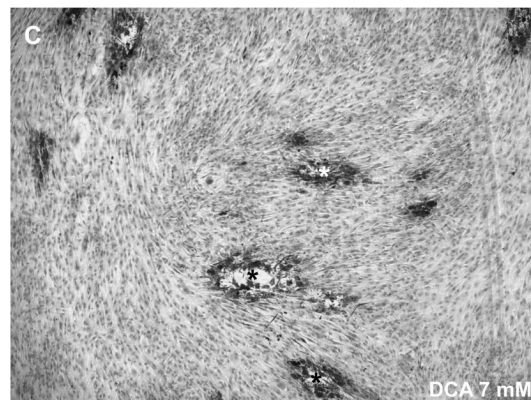

Supplement: FIG S6 [file mSphere.01002-20_sf006.pdf]
