# Supplementary material for: Dichloroacetate and Pyruvate Metabolism: Pyruvate Dehydrogenase Kinases as Targets Worth Investigating for Effective Therapy of Toxoplasmosis
Source: mSphere. 2021 Jan 6;6(1):e01002-20. doi: 10.1128/mSphere.01002-20 (PMC7845590; doi:10.1128/mSphere.01002-20)
Supplement: FIG S3 [file mSphere.01002-20_sf003.pdf]

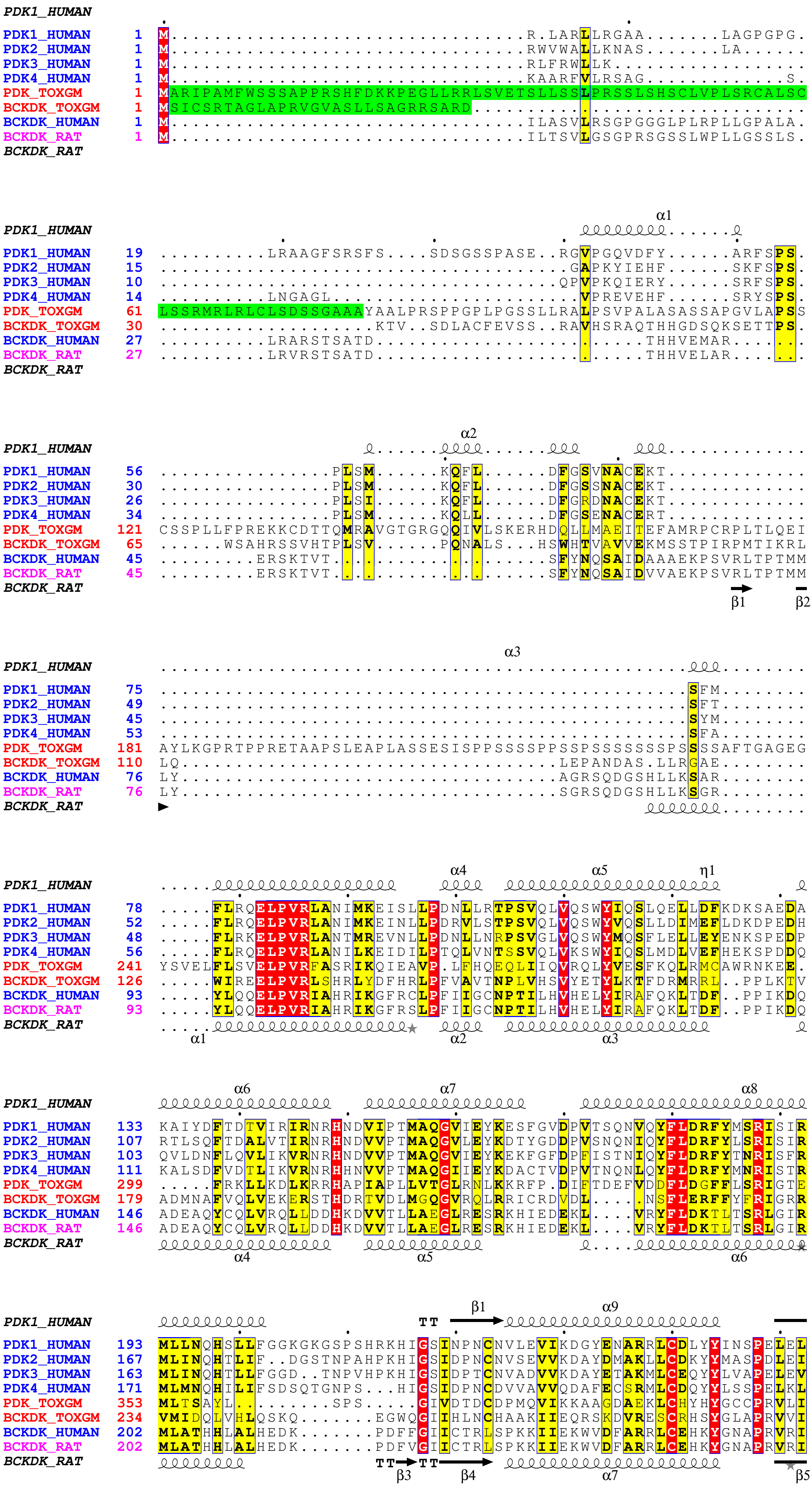

PDK1_HUMAN
PDK1_HUMAN 1 M.................................R.LARLLRGAA.......LAGPGPG.
PDK2_HUMAN 1 M.................................RWVWALLKNAS.......LA......
PDK3_HUMAN 1 M.................................RLFRWLLK..................
PDK4_HUMAN 1 M.................................KAARFVLRSAG.............S.
PDK_TOXGM 1 MARIPAMFWSSSAPPRSHFDKKPEGLLRRLSVETSLLSSLPRSSLSHSCLVPLSRCALSC
BCKDK_TOXGM 1 MSICSRTAGLAPRVGVASLLSAGRRSARD...............................
BCKDK_HUMAN 1 M.................................ILASVLRSGPGGGLPLRPLLGPALA.
BCKDK_RAT 1 M.................................ILTSVLGSGPRSGSSLWPLLGSSLS.
BCKDK_RAT
α1
PDK1_HUMAN
PDK1_HUMAN 19 ..........LRAAGFSRSFS....SDSGSSPASE..RGVPGQVDFY......ARFSPS.
PDK2_HUMAN 15 ......................................GAPKYIEHF......SKFSPS.
PDK3_HUMAN 10 .....................................QPVPKQIERY......SRFSPS.
PDK4_HUMAN 14 ..........LNGAGL.......................VPREVEHF......SRYSPS.
PDK_TOXGM 61 LSSRMRLRLCLSDSSGAAAYAALPRSPPGPLPGSSLLRALPSVPALASASSAPGVLAPSS
BCKDK_TOXGM 30 ....................KTV..SDLACFEVSS..RAVHSRAQTHHGDSQKSETTPS.
BCKDK_HUMAN 27 ..........LRARSTSATD.........................THHVEMAR.......
BCKDK_RAT 27 ..........LRVRSTSATD.........................THHVELAR.......
BCKDK_RAT
α2
PDK1_HUMAN
PDK1_HUMAN 56 ................PLSM......KQFL......DFGSVNACEKT.............
PDK2_HUMAN 30 ................PLSM......KQFL......DFGSSNACEKT.............
PDK3_HUMAN 26 ................PLSI......KQFL......DFGRDNACEKT.............
PDK4_HUMAN 34 ................PLSM......KQLL......DFGSENACERT.............
PDK_TOXGM 121 CSSPLLFPREKKCDTTQMRAVGTGRGQQIVLSKERHDQLLMAEITEFAMRPCRPLTLQEI
BCKDK_TOXGM 65 ......WSAHRSSVHTPLSV......PQNALS...HSWHTVAVVEKMSSTPIRPMTIKRL
BCKDK_HUMAN 45 ....ERSKTVT....................SFYNQSAIDAAAEKPSVRLTPTMM
BCKDK_RAT 45 ....ERSKTVT....................SFYNQSAIDVVAEKPSVRLTPTMM
BCKDK_RAT
β1 β2
α3
PDK1_HUMAN
PDK1_HUMAN 75 ....SFM........
PDK2_HUMAN 49 ....SFT........
PDK3_HUMAN 45 ....SYM........
PDK4_HUMAN 53 ....SFA........
PDK_TOXGM 181 AYLKGPRTPPRETAAPSLEAPLASSESISPPSSSSPPSSPSSSSSSSPSSSSAFTGAGEG
BCKDK_TOXGM 110 LQ...................................LEPANDAS.LLRGAE........
BCKDK_HUMAN 76 LY...................................AGRSQDGSHLLKSAR........
BCKDK_RAT 76 LY...................................SGRSQDGSHLLKSGR........
BCKDK_RAT
α4 α5 η1
PDK1_HUMAN
PDK1_HUMAN 78 .....FLRQELPVRLANIMKEISLLPDNLLRTPSVQLVQSWYIQSLQELLDFKDKSAEDA
PDK2_HUMAN 52 .....FLRQELPVRLANIMKEINLLPDRVLSTPSVQLVQSWYVQSLLDIMEFLDKPEDH
PDK3_HUMAN 48 .....FLRKELPVRLANTMREVNLLPDNLLNRPSVGLVQSWYMQSFLELLEYENKSPEDP
PDK4_HUMAN 56 .....FLRQELPVRLANILKEIDILPTQLVNTSSVQLVKSWYIQSLMDLVEFHEKSPDDQ
PDK_TOXGM 241 YSVELFLSVELPVRFASRIKQIEAVP.LFHQEQLIIQVRQLYVESFKQLRMCAWRNKEE.
BCKDK_TOXGM 126 .....WIREELPVRLSHRLYDFHRLPFVAVTNPLVHSVYETYLKTFDRMRRL..PPLKTV
BCKDK_HUMAN 93 .....YLQQELPVRIAHRIKGFRCLPFIIGCNPTILHVHELYIRAFQKLTDF..PPIKDQ
BCKDK_RAT 93 .....YLQQELPVRIAHRIKGFRSLPFIIGCNPTILHVHELYIRAFQKLTDF..PPIKDQ
BCKDK_RAT
α1 α2 α3
α6 α7 α8
PDK1_HUMAN
PDK1_HUMAN 133 KAIYDFTDTVIRIRNRHNDVIPTMAQGVIEYKESFGVDPVTSQNVQYFLDRFYMSRISIR
PDK2_HUMAN 107 RTLSQFTDALVTIRNRHNDVVPTMAQGVLEYKDTYGDDPVSNQNIQYFLDRFYLSRISIR
PDK3_HUMAN 103 QVLDNFLQVLIKVRNRHNDVVPTMAQGVIEYKEKFGFDPFISTNIQYFLDRFYTNRISFR
PDK4_HUMAN 111 KALSDFVDTLIKVRNRHHNVVPTMAQGIIEYKDACTVDPVTNQNLQYFLDRFYMNRISTR
PDK_TOXGM 299 .....FRKLLKDLKRRHAPIAPLVTGLRNLKKRFP.DIFTDEFVDDFLDGFFLSRIGTE
BCKDK_TOXGM 179 ADMNAFVQLVEKERSTHDRTVDLMGQGVRQLRRICRDVDL.....NSFLERFFYFRIGRR
BCKDK_HUMAN 146 ADEAQYCQLVRQLLDDHKDVVTLAEGLRESRKHIEDEKL....VRYFLDKTLTSRLGIR
BCKDK_RAT 146 ADEAQYCQLVRQLLDDHKDVVTLAEGLRESRKHIEDEKL....VRYFLDKTLTSRLGIR
BCKDK_RAT
α4 α5 α6
β1 α9
PDK1_HUMAN
TT
PDK1_HUMAN 193 MLLNQHSLLFGGKGKGSPSHRKHIGSINPNCNVLEVIKDGYENARRLCDLYYINSPELEL
PDK2_HUMAN 167 MLINQHTLIF..DGSTNPAHPKHIGSIDPNCNVSEVVKDAYDMAKLLCDKYYMASPDLEI
PDK3_HUMAN 163 MLINQHTLLFGGD..TNPVHPKHIGSIDPTCNVADVVKDAYETAKMLCEQYYLVAPELEV
PDK4_HUMAN 171 MLMNQHILIFSDSQTGNPS...HIGSIDPNCDVVAVVQDAFECSRMLCDQYYLSSPELKL
PDK_TOXGM 353 MLTSAYL..........SPS.....GIVDTDCDPMQVIKKAAGDAEKLCHYHYGCCPRVLI
BCKDK_TOXGM 234 VMIDQLVHLQSKQ......EGWQGIIHLNCHAAKIIEQRSKDVRESCRHSYGLAPRVVI
BCKDK_HUMAN 202 MLATHHLALHEDK.......PDFFGIICTRLSPKKIIEKWVDFARRLCEHKYGNAPRVRI
BCKDK_RAT 202 MLATHHLALHEDK.......PDFVGIICTRLSPKKIIEKWVDFARRLCEHKYGNAPRVRI
BCKDK_RAT
TT TT
β3 β4 α7 β5

```
PDK1_HUMAN          β2          TT      β3            α10
PDK1_HUMAN    253  E.ELNAKSPGQPIQVVYVPSHLYHMVFELFKNAMRATMEH................HA..
PDK2_HUMAN    225  Q.EINAANSKQPIHMVYVPSHLYHMLFELFKNAMRATVES................HE..
PDK3_HUMAN    221  E.EFNAKAPDKPIQVVYVPSHLFHMLFELFKNSMRATVEL................YE..
PDK4_HUMAN    228  T.QVNGKFPDQPIHIVYVPSHLHHMLFELFKNAMRATVEH................QE..
PDK_TOXGM     399  WNNERERF.......ACVPQYLYYILFELFKNAMRATVERFGADSSSRRSRSSAFDEEAG
BCKDK_TOXGM   287  SGNTDMKFA.......TIPDHLALIVTEVLKNALRATVEF................HTMG
BCKDK_HUMAN   255  NGHVAARFP.......FIPMPLDYILPELLKNAMRATMES................H...
BCKDK_RAT     255  NGHVAARFP.......FIPMPLDYILPELLKNAMRATMES................H...
BCKDK_RAT          TT      β6                α8

PDK1_HUMAN                                                              β4
PDK1_HUMAN    294  .............................................NRGV.YPPIQVHVTL
PDK2_HUMAN    266  .............................................SSLI.LPPIKVMVAL
PDK3_HUMAN    262  .............................................DRKEGYPAVKTLVTL
PDK4_HUMAN    269  .............................................NQ.PSLTPIEVIVVL
PDK_TOXGM     452  KSFSGVRTSRGSVGNAFSLKREEDYEYDSSFLFYGRPPQYETKLADSDMQLPPIQLVVSG
BCKDK_TOXGM   324  NSLVDA.TTRG................................LIQEDEDLPEVKVEVYK
BCKDK_HUMAN   289  ............................................LDTPYNVPDVVITIAN
BCKDK_RAT     289  ............................................LDTPYNVPDVVITIAN
BCKDK_RAT                                                  TT  TT       β7

PDK1_HUMAN            β5           α11   η2
PDK1_HUMAN    308  GNEDLTVKMSDRGGGVPLRKIDRLFNYMYSTAPRPRVETSRAV.................
PDK2_HUMAN    280  GEEDLSIKMSDRGGGVPLRKIERLFSYMYSTAPTPQPGTG.GT.................
PDK3_HUMAN    277  GKEDLSIKISDLGGGVPLRKIDRLFNYMYSTAPRPSLEPTRAA.................
PDK4_HUMAN    283  GKEDLTIKISDRGGGVPLRIIDRLFSYTYSTAPTPVMDNSRNA.................
PDK_TOXGM     512  DNRVIAIKMSDQGGGVAQESIDKIWSYMYTTARPVEIGLGQSPPTVTVPDETPSPPTVGS
BCKDK_TOXGM   351  GKREVVIKISDKGGGVPPPKLQDIWSFGYSTVGDSNTKMQENSS................
BCKDK_HUMAN   305  NDVDLIIRISDRGGGIAHKDLDRVMDYHFTT.AEASTQDPRISP................
BCKDK_RAT     305  NDVDLIIRISDRGGGIAHKDLDRVMDYHFTT.AEASTQDPRISP................
BCKDK_RAT             β8                 TT

PDK1_HUMAN
PDK1_HUMAN         ............................................................
PDK2_HUMAN         ............................................................
PDK3_HUMAN         ............................................................
PDK4_HUMAN         ............................................................
PDK_TOXGM     572  LDAAPPTIPGAVGDSRRSGETGPVWDAGATDAPAVGGNTRQLCSGSRAPSLEKPLRKLDT
BCKDK_TOXGM        ............................................................
BCKDK_HUMAN        ............................................................
BCKDK_RAT          ............................................................
BCKDK_RAT

PDK1_HUMAN
PDK1_HUMAN    351  ................................................PLAGFGYGLPIS
PDK2_HUMAN    322  ................................................PLAGFGYGLPIS
PDK3_HUMAN    320  ................................................PLAGFGYGLPIS
PDK4_HUMAN    326  ................................................PLAGFGYGLPIS
PDK_TOXGM     632  EEGSPSEGLGMTETDIRSRGDTHPTPAVGASSPPTGAGSNGPSTPQVSPLAGFGCGLPLS
BCKDK_TOXGM   395  ..................................GLGENFIRS.....DMAGYGFGLPLA
BCKDK_HUMAN   348  ..................................LFGHLDMHSGAQSGPMHGFGFGLPTS
BCKDK_RAT     348  ..................................LFGHLDMHSGGQSGPMHGFGFGLPTS
BCKDK_RAT

PDK1_HUMAN         α12        β6         β7                        α13
PDK1_HUMAN    363  RLYAQYFQGDLKLYSLEGYGTDAVIYIKALSTDSIERLPVYNKAAWKHYNTNHEADDWCV
PDK2_HUMAN    334  RLYAKYFQGDLQLFSMEGFGTDAVIYLKALSTDSVERLPVYNKSAWRHYQTIQEAGDWCV
PDK3_HUMAN    332  RLYARYFQGDLKLYSMEGVGTDAVIYLKALSSESFERLPVFNKSAWRHYKTTPEADDWSN
PDK4_HUMAN    338  RLYAKYFQGDLNLYSLSGYGTDAIIYLKALSSESIEKLPVFNKSAFKHYQMSSEADDWCI
PDK_TOXGM     692  RLYASYLGGRLEILSLPFHGSDAYLYLNRVGDK..ERMP...PNSFPHGVTQMIRGEL..
BCKDK_TOXGM   416  RAFARYFGGDIHVQSHFGIGTDVYITLNHIG..........DKEEALYYE..........
BCKDK_HUMAN   374  RAYAEYLGGSLQLQSLQGIGTDVYLRLRHID..........GREES..............
BCKDK_RAT     374  RAYAEYLGGSLQLQSLQGIGTDVYLRLRHID..........GREES..............
BCKDK_RAT          α9         β9         β10

PDK1_HUMAN
PDK1_HUMAN    423  PSREPKDMTTFRS.A
PDK2_HUMAN    394  PSTEPKNTSTYRV.S
PDK3_HUMAN    392  PSSEPRDASKYKAKQ
PDK4_HUMAN    398  PSREPKNLAK.EVAM
PDK_TOXGM     745  .RTQEELELLGLPRD
BCKDK_TOXGM   456  ..ERPELRLEHKAPG
BCKDK_HUMAN   410  ......FRI......
BCKDK_RAT     410  ......FRI......
BCKDK_RAT
```
